# Chemical Control of CRISPR Gene Editing via Conditional Diacylation Crosslinking of Guide RNAs

**DOI:** 10.1002/advs.202206433

**Published:** 2023-02-03

**Authors:** Huajun Lei, Tianying Zeng, Xiaofang Ye, Ruochen Fan, Wei Xiong, Tian Tian, Xiang Zhou

**Affiliations:** ^1^ College of Chemistry and Molecular Sciences Key Laboratory of Biomedical Polymers of Ministry of Education The Institute of Molecular Medicine Wuhan University People's Hospital Hubei Province Key Laboratory of Allergy and Immunology Wuhan University Wuhan Hubei 430072 China

**Keywords:** crosslinking strategy, conditional control, CRISPR systems, gene editing

## Abstract

Conditional control of RNA structure and function has emerged as an effective toolkit. Here, a strategy based on a one‐step introduction of diacylation linkers and azide groups on the 2′‐OH of RNA is advance. Selected from eight phosphine reagents, it is found that 2‐(diphenylphosphino)ethylamine has excellent performance in reducing azides via a Staudinger reduction to obtain the original RNA. It is demonstrated that the enzymatic activities of Cas13 and Cas9 can be regulated by chemically modified guide RNAs, and further achieved ligand‐induced gene editing in living cells by a controllable CRISPR/Cas9 system.

## Graphical abstract

In the current study, a diacylation crosslinking strategy is developed to regulate CRISPR systems and DPPEA is used to restore the RNA structure to conditionally control gene editing when needed.

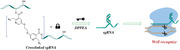

## 1 Introduction

RNAs play key roles in biological processes including gene expression, transcript splicing, messenger transcription, and translocation, as well as catalysis.^[^ ^1^ ^]^ Recent studies have pointed out other applications and properties of RNA, including RNA epigenetics,^[^ ^2^ ^]^ post‐transcription modifications,^[^ ^3^ ^]^ and chromatin regulation by long noncoding RNA.^[^ ^4^ ^]^ In these biological processes, interactions between RNA and gene expression play a crucial role at the post‐transcription level.^[^ ^3^, ^5^ ^]^ In addition, RNA‐RNA interactions are the basis for the construction of many significant regulatory networks. Due to the great complex biological roles of RNA, there is no hesitation in developing chemical toolboxes to study the functions and interactions of RNA. Chemical strategies in controlling RNA functions are applied as post‐transcriptional modifications, which include dimethyl sulfate acylation and 2′‐OH acylation (SHAPE).^[^ ^6^ ^]^ The development of chemical probing in manipulating single guide RNA (sgRNA) has prevailed in recent years. The type II CRISPR system, CRISPR‐Cas9, requires a single nuclease and one guide RNA for site recognition of specific target sequences via Watson‐Crick base pairing. Cas13a, an RNA‐guided ribonuclease, exhibits a “collateral effect” upon target recognition. Moreover, guide RNA (gRNA) is an essential part of the CRISPR system, which has been used to establish versatile methods to regulate the activity of Cas proteins. To accurately manipulate the CRISPR/Cas9 system and achieve conditionally controlled gene expression as well, many efforts have been made to broaden CRISPR/Cas9 for regulation of a specific activity, such as study based on photo‐responsive strategies, or small molecules to control Cas9 protein activity^[^ ^7^ ^]^ and gRNA structure.^[^ ^8^ ^]^ Based on CRISPR technologies, a large number of research areas have been developed, which include severe acute respiratory syndrome coronavirus 2 (SARS‐CoV‐2) detection,^[^ ^9^ ^]^ genome editing, and gene therapy.^[^ ^10^ ^]^ Various chemical crosslinking approaches have been utilized to study nucleic acid interactions, such as formaldehyde,^[^ ^11^ ^]^ carbazole derivatives,^[^ ^12^ ^]^ 4‐thiouridin,^[^ ^13^ ^]^ 1,4‐phenyldiglyoxal,^[^ ^14^ ^]^ and psoralen^[^ ^15^ ^]^ crosslinking methods. It was recently reported that bis‐nicotinic azide (BIN) probes reacted with 2′‐OH groups of different RNA strands and, via a Staudinger reduction of the azide, triggered the masking group release.^[^ ^16^ ^]^ Given the outstanding performance of this crosslinking strategy, we anticipate that this crosslinking design will promote the application of small molecules to conditionally manipulate RNA structure and then conditionally control the CRISPR systems. Conditional control of gene editing could therefore be performed through gRNA modifications with a crosslinking strategy.

**Figure 1:**
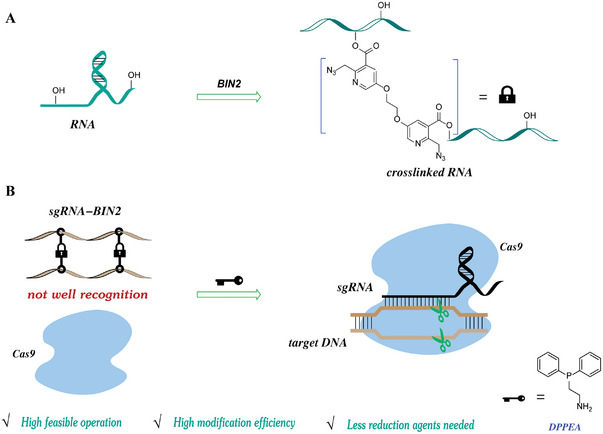
RNA crosslinking strategy for controlling CRISPR‐Cas9 gene editing in cells. A) Workflow of BIN2 probe reaction with RNA. B) Phosphine agent activation of crosslinked sgRNAs for conditional control of CRISPR gene editing in cells.

In this project, we synthesized three types of BIN compounds with different spacer lengths between the linker and reaction sites. Compared to the crosslinking efficiency of BIN2 and BIN3 in sgRNAs of the CRISPR‐Cas system, we found that BIN2 exhibited excellent performance. This prevented sgRNA from pairing with the target DNA sequence until its protective groups were detached by phosphine reduction agents upon azide reduction (**Figure** **1**). Unlike solid phase synthesis, this strategy relied on post‐transcription modifications, which can achieve modifications of large sites in RNA strands. Distinct from the acylating reagent NAI‐N_3_, here we showed that the BIN crosslinking probes had the following merits: 1) high modification efficiency, 2) low amounts of reduction agents, 3) highly feasible operations, and 4) excellent off‐to‐on switching. In addition, benefiting from the crosslinking approach of BIN2, we achieved the desired sgRNA modification and function of Cas9 in Hela cells for genome editing.

## 2 Results

### 2.1 RNA‐Crosslinking for Controlling the CRISPR‐Cas9 System

**Figure 2:**
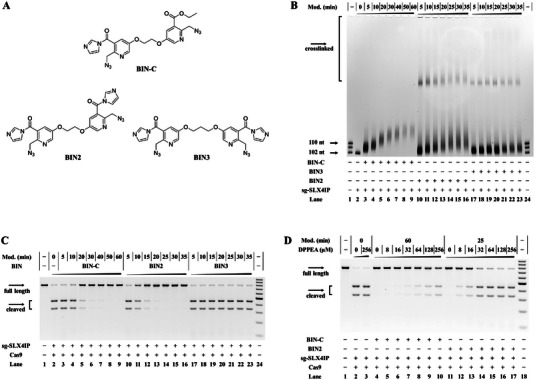
RNA crosslinking strategy for CRISPR‐Cas system regulation. Reactions were conducted as described in the Experimental Section. A) The chemical structures of BIN probes. B) Denaturing PAGE analysis of BIN probes that had crosslinked sg‐SLX4IP. Lanes 1, 24: RNA marker (sg‐SLX4IP, R‐106 nt, R‐110 nt); lane 2: untreated sg‐SLX4IP; lanes 3–9: sg‐SLX4IP modified by 25 mm BIN‐C; lanes 10–16: sg‐SLX4IP modified by 12.5 mm BIN2; lanes 17–23: sg‐SLX4IP modified by 12.5 mm BIN3. C) The influence of crosslinked sg‐SLX4IP on CRISPR‐Cas9. Lane 1: intact target DNA; lane 2: untreated sg‐SLX4IP; lanes 3–9: sg‐SLX4IP modified by 25 mm BIN‐C; lanes 10–16: crosslinked sg‐SLX4IP modified by 12.5 mm BIN2; lanes 17–23: crosslinked sg‐SLX4IP modified by 12.5 mm BIN3; lane 24: DNA marker (100‐1000 bp). D): Crosslinked sg‐SLX4IP treated with DPPEA to recover the activity of CRISPR‐Cas9. Lane 1: intact target DNA; lane 2: untreated sg‐SLX4IP; lane 3: containing untreated sg‐SLX4IP and 256 µm DPPEA; lanes 4–10: modified sg‐SLX4IP (25 mm BIN‐C, 60 min) treated with 0–256 µm DPPEA; lanes 11–17: crosslinked sg‐SLX4IP (12.5 mm BIN2, 25 min) treated with 0–256 µm DPPEA.

Inspired by previous RNA trapping methods,^[^ ^16^ ^]^ we constructed a platform for chemically‐controlled CRISPR‐Cas genome editing via an RNA crosslinking strategy in which sgRNA was modified with azide‐substituted groups (Figure 1). Numerous sgRNAs were obtained in vitro by transcription with a DNA template. We synthesized a series of compounds, including BIN‐C (one reaction active site with a 2′‐OH), BIN2 (two reaction active sites and two carbon flexible linkers), and BIN3 (two reaction active sites with three carbon flexible linkers). The synthetic route and structures of the compounds are exhibited in Schemes S1‐3 and **Figure** **2**A, respectively. Verifying the crosslinked sgRNA was vital for this study. Thus, we used denaturing polyacrylamide gel electrophoresis (PAGE) to characterize the crosslinking efficiency of the three different BIN probes with sg‐SLX4IP (the SLX4 interacting protein, *SLX4IP* gene in Figure S1, Supporting Information, and sg‐SLX4IP in Table S1, Supporting Information). sg‐SLX4IP was incubated with either BIN2 (12.5 mm), BIN3 (12.5 mm), or BIN‐C (25 mm) in SHAPE buffer (100 mm HEPES, 100 mm NaCl, and 6 mm MgCl_2_ at pH 7.5 @ 25 °C) for various durations at 37 °C. The results, shown in Figure 2B, validated that BIN2 had a higher crosslinking efficiency than BIN3. This implied that the distance of two acylating groups was significant for RNA crosslinking. Furthermore, the bis‐ and multi‐crosslinked sgRNAs migrated more slowly than the mono‐modified sgRNA (lanes 3–9 in Figure 2B), and some of the multi‐crosslinked RNAs were stagnated in the pores (lanes 10–16 in Figure 2B). Then, we observed that BIN2‐modified sg‐SLX4IP had more obvious inhibitory effects than did BIN3 or BIN‐C on the Cas9 system (Figure 2C). For BIN2 and BIN‐C, we compared the inhibition efficiency of modifying sg‐SLX4IP under different reaction times. Coupled with Cas9 protein, the DNA cleavages were terminated at either 25 or 60 min. As for BIN3, we did not observe clear inhibitory effects, which was attributed to a lack of multi‐crosslinking and a poor modification efficiency. This indicated that BIN2 was the most appropriate compound for the subsequent experiments. Next, we explored whether sg‐SLX4IP crosslinked with BIN2 could be reversed to the initial sgRNA to restore Cas9 cleavage activity. The structures of the reductants are shown in Figure S2A, Supporting Information, and from the eight candidates we found that 2‐(diphenylphosphino)ethylamine (DPPEA) displayed good performance, recovering CRISPR‐Cas9 system activity (Figure S2B, Supporting Information). We then systematically investigated the effects of DPPEA reduction on sg‐SLX4IP modified by BIN2 and BIN‐C. The results revealed that, compared with sg‐SLX4IP treated with BIN‐C, the sg‐SLX4IP crosslinked with BIN2 was sensitive to DPPEA. Treatment of 256 µm DPPEA was sufficient to drive the Cas9‐mediated cleavage of the target DNA to completion (lanes 16–17 in Figure 2D), while only 27.3% recovery was observed for sg‐SLX4IP modified by BIN‐C (lane 10 in Figure 2D). Further studies were perfromed to test the general applicability of our strategy to other types of sgRNAs, such as sg‐HBEGF (heparin‐binding EGF‐like growth factor, *HBEGF* gene in Figure S3A, Supporting Information, and results in Figure S4, Supporting Information), sg‐HPRT1 (hypoxanthine phosphoribosyl transferase 1, *HPRT1* gene in Figure S3B, and results in Figure S5, Supporting Information), and sg‐GFP (targeting different GFP fragments, t‐GFP1 and t‐GFP2 in Figure S3C,D, and results in Figure S6, Supporting Information). These results agreed with the previous findings that crosslinked sgRNAs exposed to different concentrations of DPPEA had CRISPR‐Cas9 function restored in a dose‐dependent manner.

Next, the transcribed CRISPR RNAs (crRNAs) and trans‐activating crRNA (tracrRNA) were assembled into sgRNA. To apply the crosslinking strategy in this combining system, with tracrRNA modified by BIN2, crosslinking bands were separated using denaturing PAGE (Figure S7A, Supporting Information). To further assess the inhibition effects in a Cas9 system in vitro, the agarose gel results indicated that the tracrRNA with BIN2 modifications stopped cleaving target DNA when the crosslinked tracrRNA combined with cr‐GFP RNA in a time‐dependent manner (Figure S7C,E). At the same time, we observed that the crosslinked tracrRNAs were reduced by DPPEA to reactivate Cas9 to cut the target DNA (Figure S7D,F), which was consistent with the denaturing PAGE results (Figure S7B, Supporting Information). We also tested whether cr‐SLX4IP, cr‐HPRT1, and cr‐HBEGF combined the crosslinked tracrRNA in other target DNA systems. These results agreed with the previous experiments (Figures S8–S10, Supporting Information). Notably, our crosslinking technique was used to control the CRISPR‐Cas9 system with a high reversal efficiency.

### 2.2 RNA‐Crosslinking for Controlling the RNA‐RNA Interaction

**Figure 3:**
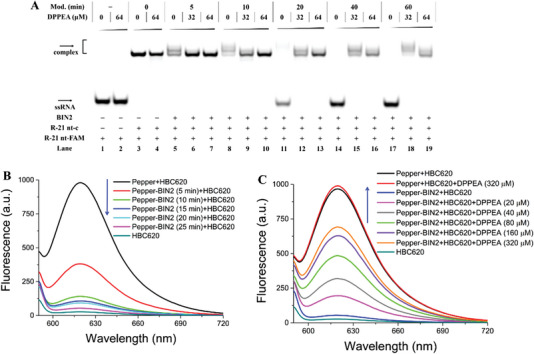
Crosslinking strategy controlling RNA structure and function. Reactions were performed as described in the Experimental Section. Products were separated on a 20% native PAGE. A) Crosslinking strategy controlling the binding of complementary RNA labeled with FAM under different treatments. Lane 1: R‐21nt‐c‐FAM; lane 2: R‐21nt‐c‐FAM and 64 µm DPPEA; lane 3: R‐21nt‐c‐FAM and complementary R‐21 nt; lane 4: R‐21nt‐c‐FAM, complementary R‐21 nt, and 64 µm DPPEA; lanes 5–7: crosslinked R‐21 nt (12.5 mm BIN2, 5 min) treated with 0–64 µm DPPEA; lanes 8–10: crosslinked R‐21 nt (12.5 mm BIN2, 10 min) treated with 0–64 µm DPPEA; lanes 11–13: crosslinked R‐21 nt (12.5 mm BIN2, 20 min) treated with 0–64 µm DPPEA; lanes 14–16: crosslinked R‐21 nt (12.5 mm BIN2, 40 min) treated with 0–64 µm DPPEA; lanes 17–19: crosslinked R‐21 nt (12.5 mm BIN2, 60 min) treated with 0–64 µm DPPEA. B) Fluorescence spectra analysis of the interaction between crosslinked Pepper (12.5 mm BIN2, 5–25 min) and HBC620. C) DPPEA activation of the crosslinked Pepper (12.5 mm BIN2, 25 min) to restore RNA function.

RNA molecules carry genetic information that plays a diverse role in biological processes.^[^ ^17^ ^]^ Subsequently, we applied the crosslinking method to 21 nt RNA strands to determine whether it had an impact on RNA binding between intermolecular RNA. Surprisingly, 21 nt RNA treated with BIN2 (crosslinked R‐21 nt) in the absence or presence of complementary R‐21nt‐c‐FAM showed that as the modification level increased, the RNA hybridization was gradually abolished (**Figure** **3**A and Figure S11A, Supporting Information). Reversible control of RNA‐RNA interactions is significant for further developing small molecule probes in complex biological processes. We next evaluated whether DPPEA removed the crosslink at 37 °C in binding buffer (10 mm Tris‐HCl and 50 mm NaCl @ 25 °C pH 7.4). Interestingly, with an increased concentration of DPPEA, two obvious binding bands appeared on the native PAGE (lanes 7, 10, 13, 16, and 19 in Figure 3A). These results showed that a conditional crosslinking design based on RNA 2′‐OH acylation was suitable for the regulation of RNA‐RNA hybridization. We also conducted circular dichroism experiments to directly demonstrate that the crosslinking approach had a tremendous impact on the binding of RNAs. Generally, the crosslinked RNA did not pair well with the complementary RNA, resulting in the melting temperature (Tm) sharply decreasing. It was observed that the RNA treated with BIN2 caused a 25.5 °C decrease in Tm compared with the untreated RNA strand (black line and magenta line in Figure S11B, Supporting Information), and that the reduced Tm value was time‐dependent. These results demonstrated that the crosslinked RNA interfered with RNA hybridization, which was in accordance with the previous native PAGE analysis.

RNAs fold into secondary or tertiary structures and participate in diverse functional regulation in cellular processes. RNA aptamers can bind fluorescent molecules in their native state, and the binding ability to dyes is sensitive to slight changes in the structure of the aptamer. The Pepper aptamer, transcribed in vitro, was selected as a model RNA strand, as it can bind the HBC chromophore in its compact state. HBC dyes were synthesized according to a previous study.^[^ ^18^ ^]^ Then, we confirmed whether the crosslinking approach worked for regulating Pepper folding and function. In our study, the chemical ligands HBC620 and HBC525 were used. For untreated Pepper, a strong green and red fluorescence signals were observed when Pepper was incubated with 2 µm HBC525 and HBC620 in a folding buffer at 37 °C for 1 h (black line in Figure 3A and Figure S12A, Supporting Information). Next, we treated Pepper with 25 mm BIN2 for 5–25 min at 37 °C in SHAPE buffer. Crosslinked Pepper lost almost all the fluorescence signal compared with the untreated samples (brown line in Figure 3B and Figure S12A, Supporting Information), implying BIN2 crosslinking completely disrupted the folded Pepper structure. We next confirmed whether the crosslinked Pepper was released when reacted with DPPEA at various concentrations at 37 °C for 2 h, followed by the addition of HBC into the reaction system for another 1 h. A recovered fluorescence signal was observed for the treated Pepper RNA (maroon line in Figure 3C and magenta line in Figure S12B, Supporting Information). These results suggest that our crosslinking strategy can be used to manipulate the structure and function of Pepper aptamers.

### 2.3 RNA‐Crosslinking for Controlling the CRISPR‐Cas13a System

**Figure 4:**
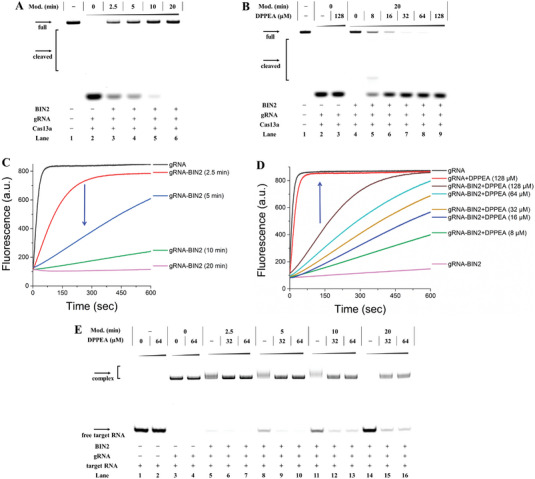
Crosslinking strategy controlling CRISPR‐Cas13a system. Reactions were conducted as described in the Experimental Section. A) Crosslinked gRNA controlling the function of the CRISPR‐Cas13a system. Lane 1: intact target RNA; lane 2: untreated gRNA; lanes 3–6: crosslinked gRNA (12.5 mm BIN2, 2.5‐20 min). B) DPPEA activation of crosslinked gRNA to recover the CRISPR‐Cas13a activity. Lane 1: intact target RNA; lane 2: untreated gRNA; lane 3: untreated gRNA and 128 µm DPPEA; lane 4: crosslinked gRNA (12.5 mm BIN2, 20 min); lanes 5–9: crosslinked gRNA (12.5 mm BIN2, 20 min) incubated with different concentrations of DPPEA to recover CRISPR‐Cas13a activity. C) The effect of crosslinked gRNA on fluorescence intensity. In this assay, gRNAs were reacted with 12.5 mm BIN2 for various time periods at 37 °C. D) DPPEA activation of crosslinked gRNA (12.5 mm BIN2, 20 min) to recover the activity of CRISPR‐Cas13a to cleave reporter RNA. E) DPPEA activation of crosslinked gRNA to bind with target RNA. Lane 1: target RNA; lane 2: target RNA and 64 µm DPPEA; lane 3: target RNA and gRNA; lane 4: target RNA, gRNA, and 64 µm DPPEA; lanes 5–7: crosslinked gRNA (12.5 mm, 2.5 min) treated with 0–64 µm DPPEA; lanes 8–10: crosslinked gRNA (12.5 mm, 5 min) treated with 0–64 µm DPPEA; lanes 11–13: crosslinked gRNA (12.5 mm, 10 min) treated with 0–64 µm DPPEA; lanes 14–16: crosslinked gRNA (12.5 mm, 20 min) treated with 0–64 µm DPPEA.

As a powerful RNA detection platform, CRISPR‐Cas13a ribonuclease can be applied to detect RNA viruses such as Zika, Dengue, and SARS‐CoV‐2.^[^ ^19^ ^]^ CRISPR‐Cas13a (previously named C2c2) is a rapid and sensitive RNA‐guided and RNA‐targeting system. To achieve artificial control of the CRISPR‐Cas13a system in vitro, we used BIN2‐modified gRNA to regulate the targeted RNA cleavage. It was anticipated that the gRNA treated with BIN2 would show a time‐dependent inhibition of RNA cleavage. The results showed that RNA cleavage was completely halted after 20 min, which implied that the crosslinking strategy markedly affected Cas13a activity (lane 6 in **Figure** **4**A). Reversible control of the Cas13a system is a key step toward using the crosslinking strategy in molecular biology. The gRNA‐BIN2 (12.5 mm, 20 min) was incubated with DPPEA, target RNA, and Cas13a at 37 °C for 2 h, after which DPPEA dose‐dependent cleavage bands were exhibited (lanes 5–9 in Figure 4B), and the entire RNA cleavage band was observed with 128 µm DPPEA. Once the Cas13a system was activated, it exhibited collateral‐cleavage activity on nonspecific RNA, causing degradation over short time periods. Here, a reporter RNA with a 5′ FAM and 3′ BHQ1 was used to perform a kinetic fluorescence assay to assess the Cas13a activity in the reporting system. Figure 4C shows that the reporter RNA was cleaved within 2 min in the untreated gRNA system. As the crosslinking level increased, we observed a decrease in fluorescence intensity due to the crosslinked gRNA not pairing well with the target RNA. Next, we asked whether DPPEA could restore gRNA structure to reactivate the Cas13a activity. The modified gRNA (12.5 mm, 20 min) reacted with DPPEA at 37 °C for 1 h, and then reporter RNA and Cas13a were added into the system, inducing an increased fluorescence signal within 12 min. In addition, 128 µm DPPEA had almost no impact on Cas13a activity. Therefore, we confirmed that our crosslinking method effectively controlled Cas13a function.

To clarify the mechanism by which the crosslinked gRNA regulated the CRISPR‐Cas13a system, we carried out a binding assay to investigate the interaction between the crosslinked gRNA and target RNA. In this experiment, gRNA containing a part of a sequence (23 nucleobases) completely paired with the targeted RNA with a 5′ FAM chromophore. We observed bands migrating more slowly than others on native PAGE, which illustrated that the unmodified gRNA bound well with the targeted RNA (lane 3 in Figure 4E and lane 2 in Figure S13, Supporting Information), and the RNA‐RNA formed complexes. We also noticed that the inhibition effects on the crosslinked gRNA and target RNA had a time‐dependent effect and completely halted binding in 20 min (lanes 5, 8, 11, and 14 in Figure 4E). De‐crosslinking is the underlying mechanism for the conditionally controlled off‐on switch of the Cas13a activity. Interestingly, the crosslinked gRNA treated with DPPEA degraded the crosslinking structure and recovered the original gRNA, and binding bands gradually reappeared. Previous studies reported that the modified gRNA still maintained its binding ability to Cas13a.^[^ ^20^ ^]^ Ultimately, we suggested that the crosslinking of gRNA, rather than the simple modification, affects the binding with Cas proteins.

### 2.4 RNA‐Crosslinking for Controlling Enzyme Recognition of Substrate

**Figure 5:**
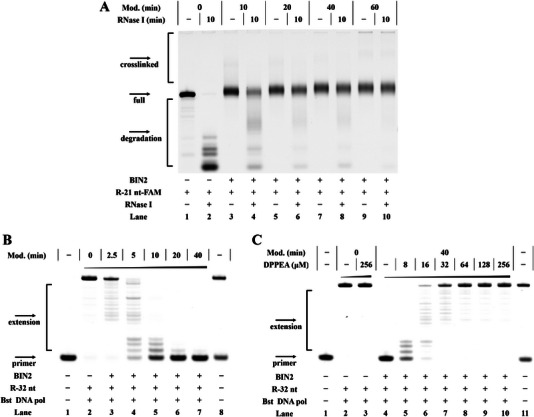
Crosslinking strategy controlling the interaction of substrate and enzyme. Reactions were performed as described in the Experimental Section. A) Crosslinked RNA resisted degradation by RNase I. Lane 1: Untreated R‐21nt‐c‐FAM; lane 2: untreated R‐21nt‐c‐FAM and RNase I; lanes 3, 5, 7, and 9: R‐21nt‐c‐FAM with different modification levels. Lanes 4, 6, 8, and 10: R‐21nt‐c‐FAM with different modification levels reacted with RNase I. B) The crosslinked RNA template inhibited primer elongation. Lane 1: DNA primer; lanes 2–7: crosslinked RNA template (12.5 mm, 0–40 min); lane 8: DNA marker. C) DPPEA activation of crosslinked RNA template to restore the activity of reverse transcriptase. Lane 1: DNA primer; lane 2: no DPPEA control; lane 3: 256 µm DPPEA treatment; lanes 4–10: crosslinked RNA template (12.5 mm BIN2, 40 min) treated with different concentrations of DPPEA (0–256 µm); lane 11: DNA marker.

RNA is a bridge between genetic material and proteins that participate in numerous cellular and biological processes. RNAs, however, are susceptible to cleavage by RNase, which is ubiquitous and inevitable in experiments. Many studies have shown that RNA with 2′‐OH chemical modification had improved stability.^[^ ^8^, ^21^ ^]^ The next significant issue was whether the crosslinked RNA had improved stability in in vitro testing. A 21 nt model RNA strand with 5′FAM labeling was utilized to examine the stability of the crosslinked RNA. In this study, two types of RNase, RNase I and RNase T1, were used in the degradation system. Representative results are shown in **Figures** **5**A and S14, Supporting Information. For untreated RNA, a series of cleaved bands were produced on 20% denaturing PAGE, which indicated that the unprotected RNA was easily degraded by RNase I and RNase T1 (lane 2 in Figures 5A and S14, Supporting Information). We then incubated the crosslinked RNA with RNases on ice for 10 min. The data revealed that, as the modification level increased, the crosslinked RNA displayed protective effects that were resistant to RNase I and RNase T1 (lanes 4, 6, 8, and 10 in Figures 5A and S14, Supporting Information). Overall, these results show that the crosslinking approach has excellent potential to protect RNA in vitro and vivo applications.

The interaction of substrate and enzyme is a basic element of enzymatic recognition and binding. RNA viruses, such as SARS‐CoV‐2 and HIV, use RNA as starting material to meet their needs.^[^ ^22^ ^]^ An important question is how the crosslinked RNA affected the interaction between the substrate and reverse transcriptase. We treated 32 nt RNA with BIN2 at 37 °C for 2.5‐40 min in SHAPE buffer. Slowly migrating bands were observed (lanes 2–6 in Figure S15A, Supporting Information). Then we tested the inhibitory effects of crosslinked RNA on the function of reverse transcription enzymes. In this system, Bst DNA polymerase (Bst DNA pol) and M‐MuLV reverse transcriptase (M‐MuLV RT) were tested. The primer extension results showed that the extent of primer extension depended on the modification levels. The RNA substrate was treated with BIN2 for 40 min, which completely eliminated the extension products (lane 8 in Figures 5B and S15C, Supporting Information). These results are related to the steric hindrance of crosslinked RNA. Next, crosslinked RNA (12.5 mm BIN2, 40 min), Bst DNA pol, primer, dNTPs, and various concentrations of DPPEA were incubated at 37 °C for 3 h, and fully extended products were observed (lane 10 in Figure 5C). Similar results were also observed with M‐MuLV RT at 42°C for 3 h (Figure S15D, Supporting Information). In addition, the denaturing PAGE results showed that crosslinked RNA reacted with 256 µm DPPEA (2 h, 37 °C) almost completely recovered the original RNA (lane 8 in Figure S15B, Supporting Information).

### 2.5 RNA‐Crosslinking for Controlling Gene Editing in Human Cells

Over results revealed that our crosslinking strategy successfully regulated the structure and function of sgRNA, and DPPEA reactivated the CRISPR‐Cas9 system. To further explore the ability of the crosslinked sgRNA to control genome editing in live cells, we selected Hela‐OC cells as a research target to stably express the Cas9 protein. Two endogenous genes, the heparin‐binding EGF‐like growth factor (*HBEGF*) and SLX4 interacting protein (*SLX4IP*), were chosen as the targeted genes. According to the transfection protocol, the untreated and treated sgRNA were transfected into Hela‐OC cells with Lipofectamine 3000 for 4 h and then the medium was refreshed. After 24 h, the efficiency of the gene editing (insertion/deletion (indel) mutation) was evaluated by a T7EI nuclease assay.^[^ ^23^ ^]^ Before performing conditional control gene editing in live cells, we first assessed the cytotoxicity of DPPEA to Hela‐OC cells. We observed that DPPEA caused no toxicity in cells up to 512 µm (Figure S16, Supporting Information). Under these conditions, the indel efficiency of endogenous HBEGF treated with sg‐HBEGF was 35.1%. Different levels of crosslinked sg‐HBEGF caused an inhibitory effect on CRISPR function in the cells (Figure S17A,C, Supporting Information). These results demonstrated that the crosslinking strategy reduced unwanted DNA cleavage and was in line with the in vitro CRISPR‐Cas9 results.

To further clarify the inhibitory mechanism, we performed an electrophoretic mobility shift assay (EMSA) to explore the interaction between sgRNAs and protein. The HaloTag ligand was labeled on nuclease‐deficient Cas9 protein (dCas9) to facilitate the imaging analysis. This dCas9 protein retained gRNA binding activity. The EMSA results showed that the untreated sg‐GFP with dCas9 formed RNP complexes (lane 2 in Figure S18A, Supporting Information). As the level of crosslinking increased, we noticed that quickly migrating, nonbinding bands appeared (lanes 3–9 in Figure S18A, Supporting Information). This was not surprising because the structure of the crosslinked sg‐GFP had changed. We also used sg‐SLX4IP to verify our speculation. A slower migrating band was shown in native PAGE, which illustrated that the original sg‐SLX4IP bound well with dCas9 (lane 2 in Figure S18B, Supporting Information). The crosslinked sg‐SLX4IP had no obvious binding bands, which was similar to the result of crosslinked sg‐GFP (lanes 3–9 in Figure S18B, Supporting Information). The EMSA results showed that the changed RNA structure and increased steric hindrance prevented the formation of the RNP duplex.

**Figure 6:**
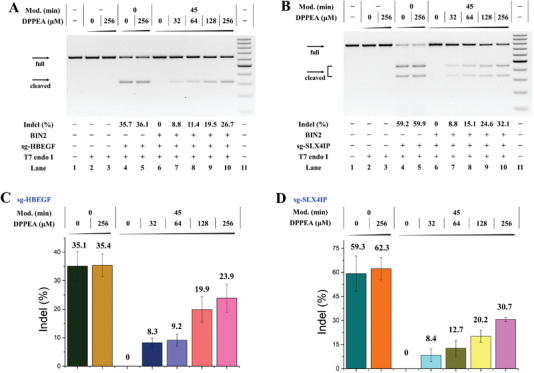
Crosslinking strategy controlling gene editing in Hela‐OC cells. Cellular studies were conducted as described in the Experimental Section. Two types of sgRNAs were transfected into Hela‐OC cells, and 4 h later DPPEA was added. After incubation for 24 h, the efficiency of gene editing rates (insertion/deletion mutation) was assessed by a T7EI nuclease assay. All samples were tested in three independent experiments. (A) Dose‐dependent activation of gene editing by DPPEA. Lane 1: target control; lane 2: no sg‐HBEGF control; lane 3: no sg‐HBEGF control and 256 µm DPPEA; lane 4: untreated sg‐HBEGF; lane 5: untreated sg‐HBEGF and 256 µm DPPEA; lanes 6–10: crosslinked sg‐HBEGF (12.5 mm BIN2, 45 min) was treated with different concentrations of DPPEA; lane 11: DNA marker (100‐1000 bp). (B) Lane 1: target control; lane 2: no sg‐SLX4IP control; lane 3: no sg‐SLX4IP control and 256 µm DPPEA; lane 4: untreated sg‐SLX4IP; lane 5: untreated sg‐SLX4IP and 256 µm DPPEA; lanes 6–10: crosslinked sg‐SLX4IP (12.5 mm BIN2, 45 min) was incubated with different concentrations of DPPEA; lane 11: DNA marker (100‐1000 bp). (C) DPPEA activation of crosslinked sg‐HBEGF was depicted in a bar graph with error bars plotted. (D) DPPEA activation of crosslinked sg‐SLX4IP was depicted in a bar graph with error bars plotted.​

To verify that the crosslinking approach regulated gene editing in a conditional manner, untreated sg‐HBEGF or crosslinked sg‐HBEGF (12.5 mm, 45 min) were transfected into Hela‐OC cells for 4 h, and then different concentrations of DPPEA were added to the system and incubated for another 24 h. A reactivated indel efficiency was exhibited by DPPEA. Compared with untreated sg‐HBEGF, the crosslinked sg‐HBEGF, treated with 256 µm DPPEA, recovered up to 70% of the gene indel rates (**Figure** **6**A,C). To further illustrate the universal applicability of this crosslinking strategy, we next targeted the *SLX4IP* gene, and the results were similar to the HBEGF results, with increased crosslinking and modification levels and a gradually decreased indel efficiency (Figure S17B,D, Supporting Information). Indel frequency was switchable by increasing the DPPEA concentration, which led to the recovery of the indel rates compared with untreated sg‐SLX4IP (≈14% for 32 µm, 20% for 64 µm, 33% for 128 µm, and 50% for 256 µm DPPEA). This caging and decaging tendency was consistent with the editing efficiency of sg‐HBEGF. Moreover, these results showed that gene editing in cells, mediated by CRISPR‐Cas9, can be conditionally regulated by the chemical activation of sgRNA.

## 3 Discussion

Regulation of RNA structure and function with small molecules provides control over cellular processes through artificial operation. Inspired by a previous study, we applied a crosslinking strategy to manipulate RNA functions, such as controlling RNA‐RNA interactions, improving RNA stability, controlling the enzyme recognition of a substrate, and regulating gene editing in live cells. Recently, CRISPR technologies have been applied in many areas, including disease detection, gene therapy, and genome editing. However, it is a great challenge for CRISPR‐Cas9 gene editing to conditionally control DNA cleavage in an efficient and conditional manner. To conquer this issue, a large number of strategies have been developed, such as optical control of Cas protein activity,^[^ ^7^ ^]^ chemical modification of gRNA,^[^ ^8^, ^24^ ^]^ and rational design of gRNA length^[^ ^25^ ^]^ and GC content.^[^ ^26^ ^]^ For our study, the chief method was based on the introduction of a diacylation linker and azide groups on the 2′‐OH of RNA, which was restored by a Staudinger reduction of the azide groups to obtain the original RNA.

In the current study, we used a diacylation crosslinking strategy to regulate the Cas protein activity and used DPPEA to restore the RNA structure to conditionally control gene editing when needed. In the CRISPR‐Cas9 system, the crosslinked sgRNA completely inhibited Cas9 protein activity. In the CRISPR‐Cas13a system, these results indicated that the untreated gRNA formed complexes with the target RNA, and the crosslinked gRNA limited the formation of the complex in a time‐dependent manner. In addition, our previous study confirmed that the modified gRNA had no effect on the formation of the binary complex between modified gRNA and dCas13a.^[^ ^8a^ ^]^ We must also mention that a considerable amount of the modified RNA products was alkylated rather than crosslinked. These results suggested these noncrosslinked products might play an important role in controlling the function of RNAs.

## 4 Conclusion

In conclusion, we have successfully established a conditional crosslinking strategy to regulate CRISPR‐Cas9 gene editing in mammalian cells. We expect that this crosslinking strategy can be further used in gene therapy and tissue engineering applications.

## 5 Experimental Section

### Materials

T4 DNA polymerase, Cas9 Nuclease, *Streptococcus pyogenes* (Cas9), Bst DNA Polymerase (Bst DNA pol), M‐MuLV Reverse Transcriptase (M‐MuLV RT), and T7 Endonuclease I were commercially available from New England Biolabs (USA). Oligonucleotide sequences containing primers are provided in Table S1 in the Supporting Information. Transcript Aid T7 High Yield Transcription kit, RNase I, and RNase T1 were purchased from Thermo Fisher Scientific. TaKaRa Shuzo Co. Ltd. (Tokyo, Japan) provided Pyrobest DNA Polymerase and PrimeSTAR HS DNA Polymerase, which were mainly used to generate target DNA. Zymo Research Corp provided DNA Clean & Concentrator‐5 kit. The nucleic acid stains Super GelRed was purchased from US Everbright Inc. (Suzhou, China). Gel Imaging was performed on Pharos FX Molecular imager and ChemiDoc MP Imaging System (Bio‐Rad, USA). The Tm measurements were executed on a JASCO‐810 spectropolarimeter (JASCO, Easton, MD, USA) equipped with a Peltier temperature controller.

### Chemical Synthesis

The target probes of BIN‐C, BIN2, and BIN3 are synthesized according to a procedure described in a previous study.^[^ ^16^ ^]^ And the synthetic routes are shown in supporting information.

### Transcription of sgRNAs

The sgRNAs forward primers and reverse primers at the aid of T4 DNA polymerase to produce DNA template, which then were transcribed in vitro by T7‐RNA polymerase at 37 °C for 3 h in transcription aid buffer, containing 20 mm MgCl_2_, 30 mm DTT, 2 mm NTPs, 2 mm spermidine, 0.1 µg µL^−1^ T7 RNA polymerase and 100 mm HEPES‐KOH pH 7.9. Through phenol/chloroform/isopropanol extraction and ethanol precipitation to obtain sgRNAs. Notes, all sgRNAs were dispersed in ultra‐pure water and preserved at −20 °C for further use.

### Crosslinking Strategy for Controlling CRISPR‐Cas9

sgRNAs were used as starting materials to prepare crosslinked sgRNAs. First, sgRNAs (3 µg); BIN‐C (25 mm), BIN2 (12.5 mm), or BIN3 (12.5 mm); and 4‐dimethylaminopyridine (DMAP, 100 mm) were reacted in SHAPE buffer at 37 °C for different periods of time, and then 90 µL ice ethanol was added to halt the reaction. Standard ethanol precipitation was used to obtain the crosslinked RNA at 100 ng µL^−1^, which was stored at −20 °C for the following experiments. Genomic DNA extracted from Hela cells was used to augment the target DNA using the following PCR primers: t‐SLX4IP‐F and t‐SLX4IP‐R for t‐SLX4IP; t‐HPRT1‐F and t‐HPRT1‐R for t‐HPRT1; and t‐HBEGF‐F and t‐HBEGF‐R for t‐HBEGF. In addition, GFP target DNA was extracted from the PEGFP‐C1 vector (Clontech) using t‐GFP‐1F and t‐GFP‐1R for t‐GFP1, and t‐GFP‐2F and t‐GFP‐2R for t‐GFP2). For the crosslinked sg‐SLX4IP‐inhibited DNA cleavage system, 40 ng of untreated sg‐SLX4IP and crosslinked sg‐SLX4IP were incubated with t‐SLX4IP (40 ng) and Cas9 (0.1 µm) in NEBuffer 3.1 (10 mm MgCl_2_, 100 mm NaCl, 50 mm Tris‐HCl, and 100 µg mL^−1^ BSA at pH 7.9 @ 25 °C) at 37°C for 6 h, then heated to 75 °C for 5 min to quench the reaction. For the DPPEA activation of the DNA cleavage system, 40 ng of untreated sg‐SLX4IP or crosslinked sg‐SLX4IP together with t‐SLX4IP (40 ng) and Cas9 (0.1 µm) were treated with DPPEA at various concentrations for 6 h, and the other procedures were similar to the previous procedures. For the sg‐HBEGF, sg‐HPRT1, and sg‐GFP systems, 50 ng untreated sgRNAs or crosslinked sgRNAs were reacted with target DNA (50 ng) and Cas9 at 37 °C for 6 h, and the subsequent steps were the same as described previously in this section.

### Crosslinking Strategy for Controlling RNA‐RNA Interaction

21 nt RNA strand at the 5′ end with FAM labeling (R‐21nt‐c‐FAM) was used to test the modified RNA whether it could bind well with the complementary RNA (R‐21 nt). The crosslinked R‐21 nt was conducted similarly to the above general protocol. For the inhibition of RNA‐RNA hybridization assay: Untreated R‐21 nt (50 ng) and crosslinked R‐21nt‐c‐FAM with different modification levels were annealed in binding buffer (50 mm KCl, 5 mm Tris‐HCl pH 6.8), and then injected on a 20% native PAGE (280 V, 2 h). For the activation of RNA‐RNA combining experiment: Crosslinked R‐21nt‐c‐FAM incubated with various concentrations of DPPEA at 37 °C for 4 h, and following the R‐21 nt was added into. The following operation is likely to be above.

### Crosslinking Strategy for Controlling CRISPR‐Cas13a

Cas13a obtained from *Leptotrichia buccalis* (LbuCas13a) was utilized in the current study. First, the crosslinked gRNA was prepared according to the previously described protocol. For the inhibition system, crosslinked gRNA (20 ng) with different modification levels was reacted with target RNA (10 ng) and Cas 13a (45 nm) in Cas13a buffer (5 mm MgCl_2_, 50 mm KCl, 5% glycerol, and 20 mm HEPES pH 6.8) at 37 °C for 1 h. The reaction was quenched by adding formamide (4.0‐fold of the reaction volume). For off‐to‐on switching of CRISPR‐Cas13a, the crosslinked gRNAs (12.5 mm, 20 ng, 20 min), target RNA (10 ng), Cas13a (45 nm), and a series of concentrations of DPPEA were incubated at 37 °C for 1 h. Fluorescence dynamic assays were used to identify the high reactivity of Cas13a. In this assay, untreated gRNA or crosslinked gRNA (100 ng) and target RNA (50 ng) were rapidly mixed with reporter RNA (200 ng) and Cas13a in 1 × Cas13a buffer. The data were collected every second at room temperature for 10 min using a fluorescence spectrophotometer (F‐4600 FL, Hitachi). For the DDPPEA conditional control reporter RNA cleavage assay, the crosslinked gRNA was incubated with various concentrations of DPPEA at 37 °C for 4 h, then the target RNA, reporter RNA, and Cas13a were added, and the data were collected every second.

### RNA Stability Assay

In vitro crosslinked RNAs stability testing were carried out as described below. 50 ng crosslinked RNAs (R‐21nt‐c‐FAM) in the RNase I buffer (20 mm Tris‐HCl, 100 mm NaCl, 0.1 mm EDTA, and 0.01% Triton X‐100, pH 8.0 @ 25°C) were incubated with RNase I (0.01 U) or RNase T1 (0.005 U) for 10 min on ice atmosphere. The reaction was quenched by the addition of a stopping mixture (4.0‐fold reaction volume of formamide and containing 0.1% SDS). Notes, the RNase T1 buffer contained 100 mm Tris‐HCl and 50 mm EDTA, pH 7.4 @ 25 °C. These enzymatic reactions were characterized by denaturing PAGE analysis (300 V, 1 h).

### Circular Dichroism (CD) Measurement

In the circular dichroism analysis assay, R‐21 nt and R‐21 nt were used, which can completely combine with each other through Watson‐Crick pairing. R‐21 nt and different levels of modification R‐21 nt were heated to 95 °C for 5 min and then cooled to room temperature naturally in CD buffer (1 mm Tris‐HCl and 5 mm NaCl, pH 7.4 @ 25 °C). Then the samples were analyzed on JASCO‐810. The signals were recorded every centigrade degree from 4 °C to 95 °C at 260 nm. Untreated R‐21 nt binding with R‐21 nt was seen as a positive control.

### Cell Culture and Cell Viability Assay

​Hela‐OC cells (stable expression Cas9 protein) were cultured in a complete medium containing 89% (v/v) basal medium (DMEM, Genview), 10% (v/v) fetal bovine serum (FBS, ExCell Bio) and 1% (v/v) pen strep (Genview) at 37 °C in a 5% CO_2_ incubator. For DPPEA toxicity analysis, Hela‐OC cells were plated 96 well plates at a density of 1.5 × 10^4^ cells per well and grown for 24 h. Then various concentrations of DPPEA along with fresh medium were added, after incubating for another 24 h and the cell viability was performed using 3‐(4,5‐dimethylthiazol‐2‐yl)‐2,5‐diphenyltetrazolium bromide (MTT). Cells were incubated with MTT (5 mg mL^−1^) for 4 h, and then the medium was discarded. Following 150 µL DMSO was added, and shaking at 70 rpm for 30 min at 37 °C. Finally, optical density was measured at 492 nm with a microplate reader (SpectraMax M5, Molecular Devices).

### Crosslinking Strategy for Controlling Gene Editing in Cells

Cells (1 × 10^5^ per well) were seeded into 24 well plates and incubated for 24 h before transferring the sgRNAs into the cells. sgRNA (500 ng µL^−1^, 1 µL per well) and DMEM (24 µL) were mixed with a solution containing Lipofectamine 3000 (1.5 µL per well, Thermo Fisher Scientific) and 23.5 µL DMEM, which were incubated for 15 min at room temperature. The transfection was started when the 50 µL mixture was added to the cells. After 4 h, cells were washed with PBS buffer and fresh complete medium was used to culture cells for another 24 h. The genome was extracted using a FastPure Cell DNA Isolation Mini Kit (Vazyme) and mutations were detected using a T7EI nuclease assay. Prior to mutation detection, the target sites were amplified using PrimeSTAR HS DNA Polymerase with primers (t‐HBEGF‐F and t‐HBEGF‐R for t‐HBEGF, or t‐SLX4IP‐F and t‐SLX4IP‐R for t‐SLXIP). The PCR product was purified by a PCR purification mini kit (TOROIVD), and 50 ng of the product was used as a substrate for T7 Endonuclease I (1.5 × 10^−3^ U for t‐SLX4IP or 3.5 × 10^−3^ U for t‐HBEGF) digestion. The reaction was performed at 37 °C for 1 h and quenched by adding loading dye containing 20 mm EDTA.

For blocking the gene editing assay, different crosslinking levels of sg‐HBEGF and sg‐SLX4IP were transfected into cells, and the rest of the procedure was similar to the previously described method.

For DPPEA activation of the gene editing assay, sgRNAs were transfected into cells for 4 h, and then cells were treated with a reduction solution containing different concentrations of DPPEA. The cells were cultured for another 24 h, and the remaining procedure was similar to the previously described method.

## Conflict of Interest

The authors declare no conflict of interest.

## Author Contributions

H.L., T.Z., X.Y. contributed equally to this work. T.T. and X.Z. conceived the original idea, designed the studies and led the project. H.J.L., X.F.Y. and R.C.F. conducted all of biological studies. T.Y.Z. synthesized BIN‐C, BIN2, and BIN3, and analyzed the NMR data. W.X. synthesized HBC620 and HBC525. All the authors provided feedback on the study and on the manuscript. The authors declare no competing financial interests.

## Supporting information

Supporting information

## Data Availability

The data that support the findings of this study are available in the supplementary material of this article.
