# Supplementary material for: Chemical Control of CRISPR Gene Editing via Conditional Diacylation Crosslinking of Guide RNAs
Source: Adv Sci (Weinh). 2023 Feb 3;10(10):2206433. doi: 10.1002/advs.202206433 (PMC10074079; doi:10.1002/advs.202206433)
Supplement: Supplementary file 1 — Supporting information [file ADVS-10-2206433-s001.pdf]

Supporting Information

Chemical control of CRISPR gene editing via conditional diacylation crosslinking of guide RNAs

Huajun Lei[‡†], Tianying Zeng[‡†], Xiaofang Ye[‡†], Ruochen Fan[†], Wei Xiong[†], Tian Tian[*,†], Xiang Zhou[†]

[†]College of Chemistry and Molecular Sciences, Key Laboratory of Biomedical Polymers of Ministry of Education, Hubei Province Key Laboratory of Allergy and Immunology, Wuhan University, Wuhan 430072, Hubei, China
[‡]Co-first author
*Correspondence: ttian@whu.edu.cn (T.T.)

This PDF file includes:

Chemical synthesis
Table of contents
Experimental section
Supplementary Table 1
Supporting Figures: S1–S19
Appendix A: NMR spectra
Appendix B: HRMS spectra
References

Chemical synthesis

Chemical reagents were purchased from Sigma-Aldrich, Heowns, and Bide. All commercially available reagents were used without further purification. Chromatographic purification was performed on silica gel (200-300 mesh). Analytical thin layer chromatography was performed using Merck Silica gel 60 F254 and visualised by UV (254 nm) ^{1}H NMR and ^{13}C NMR were recorded on 400 MHz ^{1}H (101 MHz. ^{13}C) spectrometer in deuterochloroform ($CDCl_3$). The peaks around δ 7.26 (^{1}H NMR) and 77.16 (^{13}C NMR) correspond to $CDCl_3$. Multiplicities are indicated as follows: s (singlet), d (doublet), t (triplet), m (multiplet), dd (doublet of doublets), etc. Coupling constants (J) are given in hertz. Chemical shifts (δ) are reported in parts per million relative to TMS as an internal standard. The ^{1}H and ^{13}C NMR spectra of the compounds were measured at 298 K on a 400-MHz Varian Mercury-VX400 spectrometer.

The NMR spectra of compounds are given in the appendix sections (Appendix A). And HRMS spectra of the key compounds are also exhibited (Appendix B).

Synthesis of BIN-C

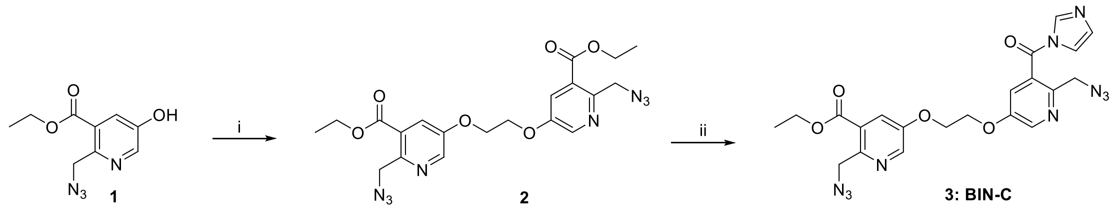

Scheme S1. Workflow of synthesis BIN-C

Synthesis of ethyl 2-(azidomethyl)-5-hydroxynicotinate (**1**)

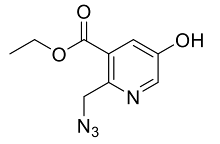

Compound 1 was synthesized according to a procedure described in previous literature.[1]

Synthesis of diethyl 5,5'-(ethane-1,2-diylbis(oxy))bis(2-(azidomethyl)nicotinate) (**2**)

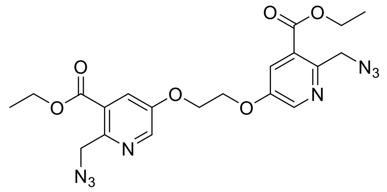

Ethylene glycol (19 mg, 0.3 mmol, 1 eq) was dissolved in dichloromethane (1 mL) and N,N-diisopropylethylamine (87 mg, 0.68 mmol, 2.2 eq) was added and the resulting mixture was cooled on ice. Triflic anhydride (182 mg, 0.64 mmol, 2.1 eq) was dissolved in dichloromethane (1 mL) and slowly added to the mixture and the resulting solution was stirred for another 1 h on ice for further using. Compound 1 (0.15 g, 0.68 mmol, 2.2 eq) and N,N-diisopropylethylamine (87 mg, 0.68 mmol, 2.2 eq) was dispersed in dichloromethane (2 mL), and then the mixture was added to above solution and stirred for 2 h at room temperature. The solution is diluted with dichloromethane and washed with 1M NaOH and then concentrated, yielding 174 mg (55%).
^{1}H NMR (400 MHz, $CDCl_3$) δ 8.49 (d, J = 3.0 Hz, 2H), 7.85 (d, J = 3.0 Hz, 2H), 4.82 (s, 4H), 4.48 (s, 4H), 4.42 (q, J = 7.1 Hz, 4H), 1.42 (t, J = 7.1 Hz, 6H).
^{13}C NMR (101 MHz, $CDCl_3$) δ 165.28, 153.88, 148.93, 140.48, 125.94, 123.49, 67.02, 62.03, 53.89, 14.19.

Synthesis of ethyl 2-(azidomethyl)-5-(2-((6-(azidomethyl)-5-(1H-imidazole-1-carbonyl)pyridin-3-yl)oxy)ethoxy)nicotinate (**3: BIN-C**)

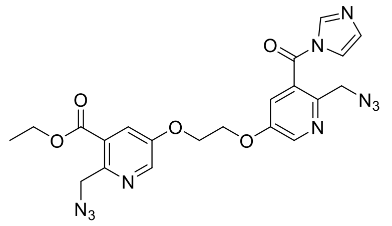

Compound 2 (174 mg, 0.38 mmol, 1 eq) was dissolved in methanol (2 mL) and 3M NaOH (272 µL, 2.2 eq) was added and the resulting solution was vigorously stirred for 16 h at room temperature. Through acidifying (1M aqueous hydrochloric acid) and extracting (ethyl acetate), the organic layer was washed with brine and dried by anhydrous Na_2SO_4. The mixture was concentrated and was further dissolved in DMF (5 mL), which was added a solution of 1,1'-carbonyldiimidazole (107 mg, 0.66 mmol, 2.2 eq) in DMF (2 mL) and the resulting mixture was stirred at room temperature for 30 min. The reaction solution was concentrated under reduced pressure, then re dissolved in ethyl acetate (60 mL) and washed with ultra-pure water for 6 times. The organic layer was concentrated under reduced pressure, yielding 138 mg (90%).

^{1}H NMR (400 MHz, $CDCl_3$) δ 8.55 (d, J = 2.8 Hz, 1H), 8.47 (d, J = 2.9 Hz, 1H), 7.94 (s, 1H), 7.83 (d, J = 2.9 Hz, 1H), 7.46 (s, 1H), 7.36 (d, J = 2.8 Hz, 1H), 7.19 (s, 1H), 4.81 (s, 2H), 4.57 (s, 2H), 4.48 (s, 4H), 4.42 (d, J = 7.1 Hz, 2H), 1.41 (t, J = 7.2 Hz, 3H).

^{13}C NMR (101 MHz, $CDCl_3$) δ 165.22, 163.88, 153.74, 153.48, 149.03, 147.40, 140.38, 140.08, 137.62, 131.88, 127.98, 125.96, 123.48, 121.54, 117.23, 67.29, 66.81, 62.05, 53.86, 53.18, 14.18.

HRMS (ESI$^+$) m/z calculated for $C_{21}H_{20}N_{10}O_5$ [M + H] $^+$: 493.1690, found: 493.1733.

Synthesis of BIN2

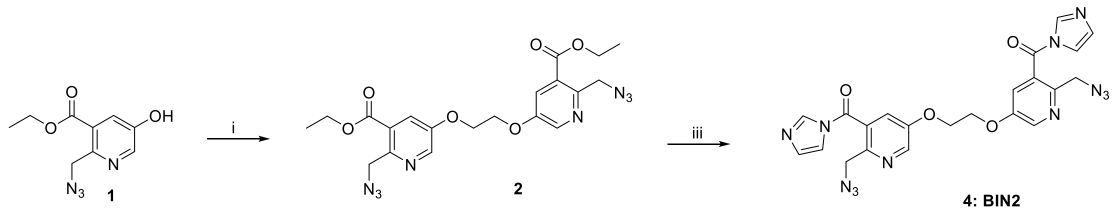

Scheme S2. Workflow of synthesis BIN2

Synthesis of ((ethane-1,2-diylbis(oxy))bis(2-(azidomethyl)pyridine-5,3-diyl))bis((1H-imidazol-1-yl)methanone) (**4: BIN2**)

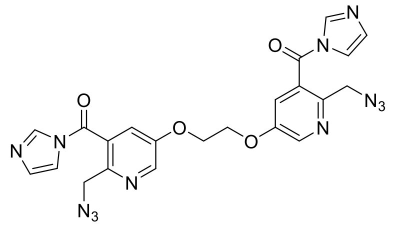

The synthesis of compound 4 is similar to compound 3 and yielding 138 mg (90%).

^{1}H NMR (400 MHz, $CDCl_3$) δ 8.54 (d, *J* = 2.9 Hz, 2H), 7.93 (s, 2H), 7.47 (s, 2H), 7.35 (d, *J* = 2.8 Hz, 2H), 7.19 (s, 2H), 4.57 (s, 4H), 4.48 (s, 4H).
^{13}C NMR (101 MHz, $CDCl_3$) δ 163.86, 153.39, 147.61, 140.02, 137.66, 131.94, 128.06, 121.63, 117.26, 67.14, 53.21.
HRMS (ESI$^+$) m/z calculated for $C_{22}H_{18}N_{12}O_4$ [M + H] $^+$: 515.1646, found: 515.1678.

Synthesis of BIN3

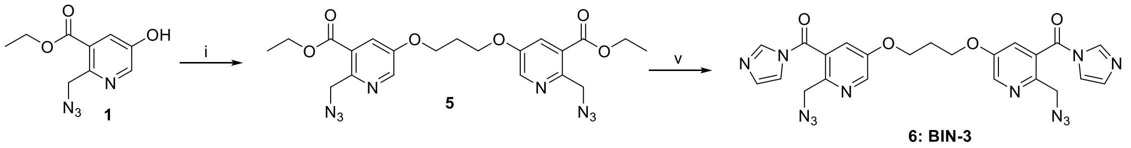

Scheme S3. Workflow of synthesis BIN3

Synthesis of diethyl 5,5'-(propane-1,3-diylbis(oxy))bis(2-(azidomethyl)nicotinate) (**5**)

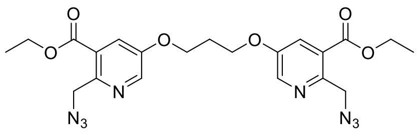

1,3-Propanediol (23 mg, 0.31 mmol, 1 eq) was dissolved in dichloromethane (1 mL) and N,N-diisopropylethylamine (87 mg, 0,68 mmol, 2.2 eq) was added and the resulting mixture was cooled on ice. Triflic anhydride (182 mg, 0.64 mmol, 2.1 eq) was dissolved in dichloromethane (1 mL) and slowly added to the reaction and the resulting solution was stirred for 1 hour on ice for following utilization. Compound 4 (150 mg, 0.68 mmol, 2.2 eq) was dissolved in dichloromethane (2 mL) and N,N-diisopropylethylamine (87 mg, 0.68 mmol, 2.2 eq) was added to the above solution and the resulting mixture was stirred for 2 h at room temperature. The solution is diluted with dichloromethane and washed with 1M NaOH and then concentrated to yield of the product (74 mg, 50%).
^{1}H NMR (400 MHz, $CDCl_3$) δ 8.44 (d, *J* = 2.9 Hz, 2H), 7.78 (d, *J* = 2.9 Hz, 2H), 4.80 (s, 4H), 4.42 (q, *J* = 7.1 Hz, 4H), 4.29 (t, *J* = 5.9 Hz, 4H), 2.41–2.31 (m, 2H), 1.42 (t, *J* = 7.1 Hz, 6H).
^{13}C NMR (101 MHz, $CDCl_3$) δ 165.40, 154.13, 148.41, 140.47, 125.93, 123.11, 64.70, 61.98, 53.91, 28.93, 14.20.
HRMS (ESI$^+$) m/z calculated for $C_{21}H_{24}N_8O_6$ [M + H] $^+$: 485.1891, found: 485.1883.

Synthesis of ((propane-1,3-diylbis(oxy))bis(2-(azidomethyl)pyridine-5,3-diyl))bis((1H-imidazol-1-yl)methanone) (**6: BIN3**)

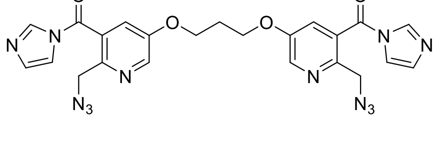

The synthesis of compound 6 is similar to compound 3 and yielding 53 mg (90%).
^{1}H NMR (400 MHz, $CDCl_3$) δ 8.49 (d, *J* = 2.8 Hz, 2H), 7.92 (s, 2H), 7.46 (s, 2H), 7.29 (d, *J* = 2.8 Hz, 2H), 7.18 (s, 2H), 4.54 (s, 4H), 4.27 (t, *J* = 5.8 Hz, 4H), 2.42 – 2.32 (m,

2H).
^{13}C NMR (101 MHz, $CDCl_3$) δ 163.93, 153.60, 147.00, 140.12, 137.64, 131.81, 127.92, 121.10, 117.22, 64.87, 53.14, 28.78.
HRMS (ESI^+) m/z calculated for $C_{23}H_{20}N_{12}O_4$ [M + H] $^+$: 529.1803, found: 529.1818.

Synthesis of HBC525 and HBC620

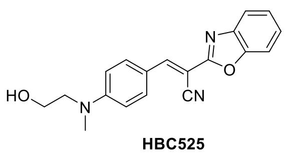

HBC525

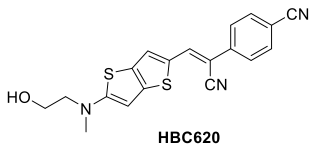

HBC620

The synthesis of HBC525 and HBC620 were conducted according to previous literatures.[2]

Table of contents

	Chemical synthesis	Page S2-6
	Table of contents	Page S7-9
	Experimental section	Page S9
**Table S1**	DNA and RNA sequences used in the current study	Page S10-11
**Figure S1**	Schematic illustration of target SLX4IP DNA sequence	Page S12
**Figure S2**	Phosphine reagents activation of CRISPR-Cas9	Page S12-13
**Figure S3**	Schematic illustration of target HBEGF DNA sequence, target HPRT1 DNA sequence, target t-GFP1 DNA sequence and target t-GFP2 DNA sequence	Page S13-14
**Figure S4**	DPPEA activation of crosslinked sg-HBEGF to reversible controlling CRISPR-Cas9	Page S14-15
**Figure S5**	DPPEA activation of crosslinked sg-HPRT1 to reversible controlling CRISPR-Cas9	Page S15-16
**Figure S6**	DPPEA activation of crosslinked sg-GFP to reversible controlling CRISPR-Cas9	Page S16-17
**Figure S7**	DPPEA activation of crosslinked tracrRNA and native crGFP to reversible controlling CRISPR-Cas9	Page S17-18
**Figure S8**	DPPEA activation of crosslinked tracrRNA and native cr-SLX4IP to reversible regulating CRISPR-Cas9	Page S18
**Figure S9**	DPPEA activation of crosslinked tracrRNA and native cr-HPRT1 to reversible controlling CRISPR-Cas9	Page S18-19
**Figure S10**	DPPEA activation of crosslinked tracrRNA and native cr-HBEGF to reversible regulating CRISPR-Cas9	Page S19
**Figure S11**	Crosslinking strategy for regulating RNA-RNA interaction	Page S19
**Figure S12**	Crosslinking strategy for controlling RNA folding	Page S20
**Figure S13**	Crosslinking strategy for regulating RNA hybridization	Page S20
**Figure S14**	Crosslinking strategy protects RNA from RNase T1 degradation	Page S20-21
**Figure S15**	DPPEA activation of crosslinked RNA template to reversible regulating primer elongation	Page S21
**Figure S16**	The influence of DPPEA to Hela-OC viability	Page S22
**Figure S17**	Crosslinking strategy controlling gene editing in Hela-OC cells	Page S22-23
**Figure S18**	Crosslinking strategy for controlling the interaction between RNA and protein	Page S23

**Appendix A**	NMR spectra of synthesized compounds	Page S24-28
**Appendix B**	HRMS spectra of the key compounds in the current study	Page S29-30
	References	Page S31

Experimental section

Crosslinking strategy for controlling Pepper aptamer folding. In this assay, RNA aptamer named Pepper was used to test the crosslinked Pepper disturbing the recognition between aptamer and fluorescence molecules (HBC620 and HBC525). Crosslinked Pepper was separately incubated with HBC525 or HBC620 (10 μM) in folding buffer (5 mM $MgCl_2$, 100 mM KCl, 40 mM HEPES, pH 7.4 @ 25°C) at 37°C for 2 h. Pepper without any treatment was used as a control in this assay. Then the fluorescence intensity was analyzed on LS-55 FL Spectrophotometer (Perkin Elmer).

For fluorescence recovery assay mediated by DPPEA, the crosslinked Pepper was firstly incubated with DPPEA of different concentrations in RNase-free water at 37°C for 4 h, and then the samples were incubated with HBC and analyzed as before.

Crosslinking strategy for controlling enzyme recognition. Bst DNA pol assay: Untreated R-32 nt (34 ng) or crosslinked R-32 nt (12.5 mM BIN2 for various of time), dNTPs, DNA primer (11 ng) 5' end with FAM labeling and Bst DNA pol (2.4 U) were incubated at 42°C for 3 h in reaction buffer (10 mM KCl,10 mM $(NH_4)_2SO_4$, 2 mM $MgSO_4$, 0.1% Triton X-100, 20 mM Tris-HCl, pH 8.8 @ 25°C). M-MuLV RT: Untreated R-32 nt (34 ng) or crosslinked R-32 nt (12.5 mM BIN2 for various of time), dNTPs, DNA primer (11 ng) 5' end with FAM labeling and M-MuLV RT (2.4 U) were incubated at 37°C for 3 h in reaction buffer (3 mM $MgCl_2$, 75 mM KCl, 50 mM Tris-HCl, pH 8.3 @ 25°C).

The recovery assays of the reverse transcription were carried out by treating crosslinked R-32 nt with different concentrations of DPPEA at 37°C for 4 h, and the following procedures are similar to before.

Cas13a, dCas9 and dCas13a of expression and purification. This part of the experiment procedure is similar to the recently published studies.[3]

Supplementary Table 1. DNA and RNA sequences used in the current study

Name	Sequence (from 5' to 3')	Construct
R-32 nt	5'-AAGUCGAUCUCAGUGCAGUACAAGUAAUCCAU-3'	RNA
primer	5'-FAM-ATGGATTACTT-3'	DNA primer
R-21nt-c	5'-AAGCUGAUCUCGAUGACGUAC-3'	RNA
R-21nt	5'-GUACGUCAUCGAGAUCAGCUU-3'	RNA
R-21nt-c-FAM	5'-FAM-AAGCUGAUCUCGAUGACGUAC-3'	RNA
gRNA	5'-GAUUUAGACUACCCCAAAAACGAAGGGGACUAAAA CUAGAUUGCUGUUCUACCAAGUAAUCCAU-3'	RNA
target RNA	5'-FAM-UUACUUGGUAGAACAGCAAUCUA-3'	RNA
reporter RNA	5'-FAM-UUUUU-BHQ1-3'	RNA
sg-SLX4IP-F	5'-TCTAATACGACTCACTATAGGGCCACAGCCAGGATTTAA GAGTTTTAGAGCTAGAAATAGCAAGTTAAAATA-3'	The forward primer for sg-SLX4IP construct
sg-HBEGF-F	5'-TCTAATACGACTCACTATAGGGTTCTCTCGGCACTGGTGA CGTTTTAGAGCTAGAAATAGCAAGTTAAAATA-3'	The forward primer for sg-HBEGF construct
sg-HPRT1-F	5'-TCTAATACGACTCACTATAGGGCCCAAGGAAAGACTATGA AAGTTTTAGAGCTAGAAATAGCAAGTTAAAATA-3'	The forward primer for sg-HPRT1 construct
sg-GFP-F	5'-TCTAATACGACTCACTATAGGGATGCCGTTCTTCTGCTTG TGTTTTAGAGCTAGAAATAGCA-3'	The forward primer for construct sg-GFP
(sg-GFP + 4)-F	5'-TCTAATACGACTCACTATAGGGATGCATGCCGTTCTTCTG CTTGTGTTTTAGAGCTAGAAATAGCA-3'	The forward primer for construct sg-GFP + 4
(sg-GFP + 8)-F	5'-TCTAATACGACTCACTATAGGGATGCATGCATGCCGTTCT TCTGCTTGTGTTTTAGAGCTAGAAATAGCA-3'	The forward primer for construct sg-GFP + 8
sg-R	5'-AAAAGCACCGACTCGGTGCCACTTTTTCAAGTTGATAAC GGACTAGCCTTATTTTAACTTGCTATTTCTA-3'	The reverse primer for sgRNA construct
sg-GFP-R	5'-AAAAGCACCGACTCGGTGCCACTTTTTCAAGTTGATAAC GGACTAGCCTTATTTTAACTTGCTATTTCTAGCTCTAAAAC-3'	The reverse primer for sg-GFP, sg-GFP+4, sg-GFP+8 construct
tracrRNA-F	5'-TCTAATACGACTCACTATAGGGTTGGAACCATTCAAAACA GCATAGCAAGTTAAAATAAGGCTAG-3'	for tracrRNA construct
tracrRNA-R	5'-AAAAAAAGCACCGACTCGGTGCCACTTTTTCAAGTTGAT AACGGACTAGCCTTATTTTAACTTGCT-3'	
sg-SLX4IP	5'-GGGCCACAGCCAGGAUUUAAGAGUUUUAGAGCUAGAA AUAGCAAGUUAAAAUAAGGCUAGUCCGUUAUCAACUUGA AAAAGUGGCACCGAGUCGGUGCUUUU-3'	transcribed RNA
sg-HBEGF	5'-GGGUUCUCUCGGCACUGGUGACGUUUUAGAGCUAGAA	transcribed RNA

	AUAGCAAGUUAAAAUAAGGCUAGUCCGUUAUCAACUUGA AAAAGUGGCACCGAGUCGGUGCUUUU-3’	
sg-HPRT1	5’-GGGCCCAAGGAAAGACUAUGAAAGUUUUAGAGCUAGA AAUAGCAAGUUAAAAUAAGGCUAGUCCGUUAUCAACUUG AAAAAGUGGCACCGAGUCGGUGCUUUU-3’	transcribed RNA
sg-GFP (102 nt)	5’-GGGAUGCCGUUCUUCUGCUUGUGUUUUAGAGCUAGAA AUAGCAAGUUAAAAUAAGGCUAGUCCGUUAUCAACUU GAAAAAGUGGCACCGAGUCGGUGCUUUU-3’	transcribed RNA
sg-GFP+4 (106 nt)	5’-GGGAUGCAUGCCGUUCUUCUGCUUGUGUUUUAGAGCU AGAAAUAGCAAGUUAAAAUAAGGCUAGUCCGUUAUCAAC UUGAAAAAGUGGCACCGAGUCGGUGCUUUU-3’	transcribed RNA
sg-GFP+8 (110 nt)	5’-GGGAUGCAUGCAUGCCGUUCUUCUGCUUGUGUUUUAG AGCUAGAAAUAGCAAGUUAAAAUAAGGCUAGUCCGUUAU CAACUUGAAAAAGUGGCACCGAGUCGGUGCUUUU-3’	transcribed RNA
tracrRNA	5’-GGGUUGGAACCAUUCAAAACAGCAUAGCAAGUUAAAA UAAGGCUAGUCCGUUAUCAACUUGAAAAAGUGGCACCGA GUCGGUGCUUUUUUU-3’	transcribed RNA
cr-SLX4IP	5’-GGGCCACAGCCAGGAUUUAAGAGUUUUAGAGCUAUGC UGUUUUG-3’	RNA
cr-HBEGF	5’-GGGUUCUCUCGGCACUGGUGACGUUUUAGAGCUAUGC UGUUUUG-3’	RNA
cr-HPRT1	5’-GGGCCCAAGGAAAGACUAUGAAAGUUUUAGAGCUAUG CUGUUUUG-3’	RNA
cr-GFP	5’-GGGAUGCCGUUCUUCUGCUUGUGUUUUAGAGCUAUGC UGUUUUG-3’	RNA
t-SLX4IP-F	5’-TTATCC GGCACT GTG AAAGCT-3’	For PCR of t-SLX4IP
t-SLX4IP-R	5’-CCTGAT GTTTAG CAAC TTTTTGG-3’	
t-HBEGF-F	5’-GCCGCTTCGAAAGTGACTGG-3’	For PCR of t-HBEGF
t-HBEGF-R	5’-GATCCCCCAGTGCCCATCAG-3’	
t-HPRT1-F	5’-GTTGTGATAAAAGGTGATGCTC-3’	For PCR of t-HPRT1
t-HPRT1-R	5’-TCATAAACACATCCATGGGAC-3’	
t-GFP-1F	5’-GAGGAGCTGTTCACCGGG-3’	For PCR of t-GFP1
t-GFP-1R	5’-CTTGTACAGCTCGTCCATGC-3’	
t-GFP-2F	5’-GACGTAAACGGCCACAAGTTC-3’	For PCR of t-GFP2
t-GFP-2R	5’-GGGGTGTTCTGCTGGTAGTG-3’	

TTATCCGGCACTGTGAAAGCTACCTTTCCTTTCTCACAATCTTTCGCACCGAATATAT
GTTCCATTCTTGAGCTAGTCATTTTCCAATGTTAAGAAAACACACCACAGAAAAGCGTT
TTTTTTTCTCTCGGTCAGTTCAACTGCCTAGCACCGTGAGTTACCATGATACGTTATTTG
CTAATGAGAGTCATTGGGGTCCCAGCCTTTAAAAATAGCCTGCGTCCGGCGCCATTTTT
GTTTTGGGCCCACTTTGAAAGCCGCGATATTCTGAACAGAGGCCATTTTTCTTTTGGGC
AGATTCCACGGGCCATCGAAGTGTTTCGCTGCCA**TCT*TAAATCCTGGCTGTGGC**CTC
AATCCTGCAGCGAAGCTGCCCAGATCATTTTGTGTTTGGGCGGCGGCTGAGTTCCCAA
ACGCGCGCGGAGACCCTGGGAAGGAGGAAGTAAGCGCGAAAGTGCTTCCCTTAAGCT
TCTGAAGGTTGGCTGCAGTTCCGGCTACCTGTGTAGTCCGAGTTTCCACAGCCAGGTA
CTACTCCGCCAGTGACCCTGGACAGTAACAAAACATATAAAGCCCGAGCCCAAACCCC
GCCACCATCATAGGTAAGCACATGGACCTCTGACAACCTCAGATGTTCCTTCAAGTGA
ACTGAACTGTTTGCCCATCTCCTCCCTCCTGGTTTTTGGCTTTGATTTTTTGAGACTTTT
CCGAATCCTTCTCCCTGTACTTCCACCTTTCTTCATTGTTTTCAAAAAG**CCAAAAAGTT**
GCTAAACATCAGG

Figure S1. **Schematic illustration of target SLX4IP DNA sequence**. The target site and PCR primer were indicated by green color and underlining, respectively. Red asterisk displayed the cleavage sites by Cas9 nuclease. Schematic illustration of the sequence of *SLX4IP* gene around target loci. The *SLX4IP* gene is located on the short arm (p) of chromosome 20 at position 12.2 (20p12.2). We generated target SLX4IP DNA (t-SLX4IP) carrying the target loci from Hela-OC genomic DNA. The 20 nt sequence was the same sequence as the target sequence.

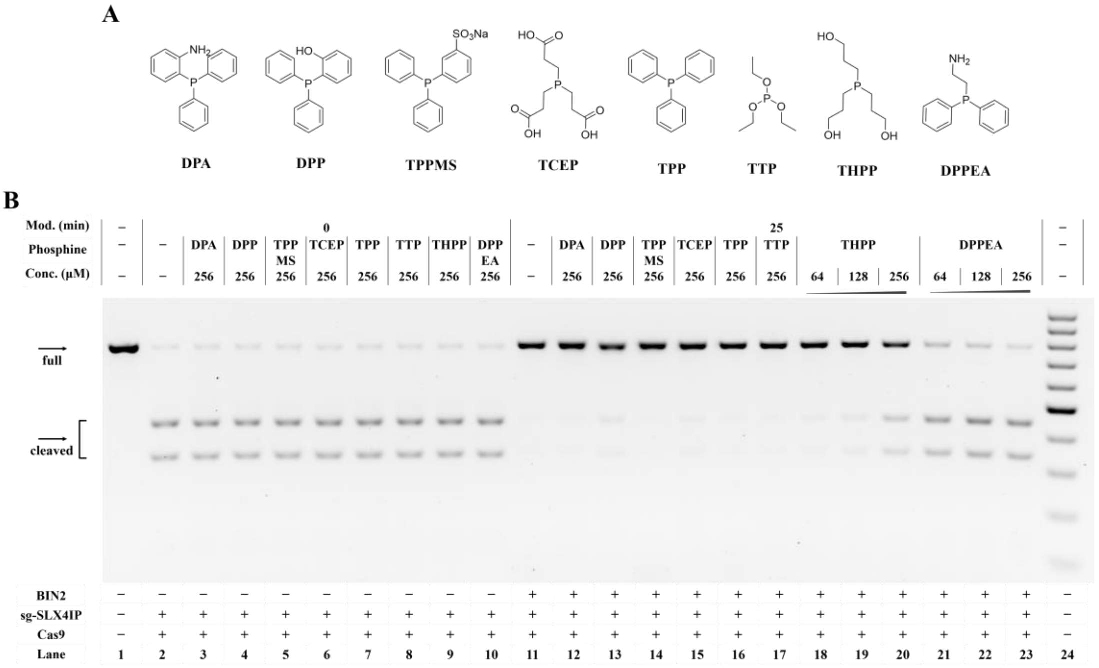

Figure S2. Phosphine reagents activation of CRISPR-Cas9. Reactions were performed as described in the Experimental Section. (A) Chemical structure of phosphine reagents. The used phosphines were exhibited here. (B) Phosphine reagents activation crosslinked sg-SLX4IP to reversible control DNA cleavage by Cas9. Lane 1: intact DNA; lane 2: untreated sg-SLX4IP; lanes 3–10: untreated sg-SLX4IP and DPA, DPP, TPPMS, TCEP, TPP, TTP, THPP and DPPEA, respectively; lane 11: crosslinked sg-SLX4IP (12.5 mM BIN2, 25 min); lanes 11–17: crosslinked sg-SLX4IP (12.5 mM BIN2, 25 min) incubated with 256 μM DPA, DPP, TPPMS, TCEP, TPP and TTP; lanes 18–20: crosslinked sg-SLX4IP (12.5 mM BIN2, 25 min) incubated with THPP; lanes 21–23: crosslinked sg-SLX4IP (12.5 mM BIN2, 25 min) incubated with DPPEA; lane 24: DNA marker.

A

GCCGCTTCGAAAGTGACTGGTGCCTCGCCGCCTCCTCTCGGTGCGGGACCATGAAG
CTGCTGCCGTCGGTGGTGCTGAAGCTCTTTCTGGCTGCAGGTAAGAGGGCTGCCGACG
CCCCCGGAGATCGGGGGGATGGGGGCGTTGTGCTGGGGGCATGGGGGAAGGTCGCCG

CAGCGCACCCGGCACGGGCCACTTGGTGGGGCCCTTGCGCTCTGGCGGACGGGCGTC
GGCATCGGTGCGTGTTGGTCAGGGGTCTGGGCGGGTGTCTGATGCGGCCTGGCCTCTC
GCCCGCAG**TTCTCTCGGCACTGGT*GAC**TGGCGAGAGCCTGGAGCGGCTTCGGAGA
GGGCTAGCTGCTGGAACCAGCAACCCGGACCCTCCCACTGTATCCACGGACCAGCTGC
TACCCCTAGGAGGCGGCCGGGACCGGAAAGTCCGTGACTTGCAAGAGGCAGATCTGG
ACCTTTTGAGAGGTGGGTGTGGAGGCCCCCCATCCTTGGACCTTGGTGGGCTGTTGAA
GAATAAGCAGATCCAAGATTCTTGCTGTTTGGGCAATACTGTGGGTTGAGGGTATTCAT
GGAGAACCTCGGGGAAAAGCTGATCGGC**CTGATGGGCACTGGGGGATC**

B

>X dna:chromosome chromosome:GRCh38:X:134460165:134520513:1

GTTGTGATAAAAGGTGATGCTCACCTCTCCCACACCCTTTTATAGTTTAGGGATTGTA
TTTCCAAGGTTTCTAGACTGAGAGCCCTTTTCATCTTTGCTCATTGACACTCTGTACCC
ATTAATCCTCCTTATTAGCTCCCCTTCAATGGACACATGGGTAGTCAGGGTGCAGGTCT
CAGAACTGTCCTTCAGGTTCCAGGTGATCAACCAAGTGCCTTGTCTGTAGTGTCAACT
CATTGCTGCCCCTTCCTAGTAATCCCCATAATTTAGCTCTCCA**TTT*CATAGTCTTTCCT**
TGGGTGTGTTAAAAGTGACCATGGTACACTCAGCACGGATGAAATGAAACAGTGTTTA
GAAACGTCAGTCTTCTCTTTTGTAATGCCCTGTAGTCTCTCTGTATGTTATATGTCACATT
TTGTAATTAACAGCTTGCTGGTGAAAAGGACCCCACGAAGTGTTGGATATAAGCCAGA
CTGTAAGTGAATTACTTTTTTTGTCAATCATTTAACCATCTTTAACCTAAAAGAGTTTTAT
GTGAAATGGCTTATAATTGCTTAGAGAATATTTGTAGAGAGGCACATTTGCCAGTATTAG
ATTTAAAAGTGATGTTTTCTTTATCTAAATGATGAATTATGATTCTTTTTAGTTGTTGGAT
TTGAAATTCCAGACAAGTTTGTTGTAGGATATGCCCTTGACTATAATGAATACTTCAGGG
ATTTGAATGTAAGTAATTGCTTCTTTTTCTCACTCATTTTTCAAAACACGCATAAAAATT
TAGGAAAGAGAATTGTTTTCTCCTTCCAGCACCTCATAATTTGAACAGACTGATGGTTC
CCATTAGTCACATAAAGCTGTAGTCTAGTACAGACGTCCTTAGAACTGGAACCTGGCCA
GGCTAGGGTGACACTTCTTGTTGGCTGAAATAGTTGAACAGCTTTAATATACAATAATT
GTTGCATTATTATTTCAGATGATAAATGTGGTCATAAGTAAGAAATAAATGATCGAGTTT
AGTCTTTTAATTCACTGTCCTTTGAATACCTGCCTCTTACTCTGGAGGCAGAA**GTCCCA**
TGGATGTGTTTATGA

C

GAGGAGCTGTTCACCGGGGTGGTGCCCATCCTGGTCGAGCTGGACGGCGACGTAAA
CGGCCACAAGTTCAGCGTGTCCGGCGAGGGCGAGGGCGATGCCACCTACGGCAAGCT
GACCCTGAAGTTCATCTGCACCACCGGCAAGCTGCCCGTGCCCTGGCCCACCCTCGTG
ACCACCCTGACCTACGGCGTGCAGTGCTTCAGCCGCTACCCCGACCACATGAAGCAGC
ACGACTTCTTCAAGTCCGCCATGCCCGAAGGCTACGTCCAGGAGCGCACCATCTTCTT
CAAGGACGACGGCAACTACAAGACCCGCGCCGAGGTGAAGTTCGAGGGCGACACCCT
GGTGAACCGCATCGAGCTGAAGGGCATCGACTTCAAGGAGGACGGCAACATCCTGGG
GCACAAGCTGGAGTACAACTACAACAGCCACAACGTCTATATCATGGCCG**ACAAGCA**
GAAGAACGGCA*TCAAGGTGAACTTCAAGATCCGCCACAACATCGAGGACGGCAGC
GTGCAGCTCGCCGACCACTACCAGCAGAACACCCCCATCGGCGACGGCCCCGTGCTG
CTGCCCGACAACCACTACCTGAGCACCCAGTCCGCCCTGAGCAAAGACCCCAACGAG
AAGCGCGATCACATGGTCCTGCTGGAGTTCGTGACCGCCGCCGGGATCACTCTCG**GCA**
TGGACGAGCTGTACAAG

D

GACGTAAACGGCCACAAGTTCAGCGTGTCCGGCGAGGGCGAGGGCGATGCCACCTA
CGGCAAGCTGACCCTGAAGTTCATCTGCACCACCGGCAAGCTGCCCGTGCCCTGGCCC
ACCCTCGTGACCACCCTGACCTACGGCGTGCAGTGCTTCAGCCGCTACCCCGACCACA
TGAAGCAGCACGACTTCTTCAAGTCCGCCATGCCCGAAGGCTACGTCCAGGAGCGCA
CCATCTTCTTCAAGGACGACGGCAACTACAAGACCCGCGCCGAGGTGAAGTTCGAGG
GCGACACCCTGGTGAACCGCATCGAGCTGAAGGGCATCGACTTCAAGGAGGACGGCA
ACATCCTGGGGCACAAGCTGGAGTACAACTACAACAGCCACAACGTCTATATCATGGC
CG**ACAAGCAGAAGAACGGCA*TC**AAGGTGAACTTCAAGATCCGCCACAACATCGAG
GACGGCAGCGTGCAGCTCGCCGAC**CACTACCAGCAGAACACCCC**

Figure S3. **Schematic illustration of target HBEGF DNA sequence, target HPRT1 DNA sequence, target t-**

GFP1 DNA sequence and target t-GFP2 DNA sequence. The target site and PCR primer were indicated by green color and underlining, respectively. Red asterisk displayed the cleavage sites by Cas9 nuclease. (**A**) Schematic illustration of the 5′-UTR sequence of *HBEGF* gene around target loci. We obtained target HBEGF DNA (t-HBEGF) carrying the target loci from Hela-OC genomic DNA. The 20 nt sequence was the same sequence as the target sequence. (**B**) Schematic illustration of the sequence of *HPRT1* gene around target loci. We obtained target HPRT1 DNA (t-HPRT1) carrying the target loci from Hela-OC genomic DNA. The 20 nt sequence was the exact same sequence as the target sequence. Additionally, we obtained two different GFP targets (t-GFP1 and t-GFP2) carrying the target loci from pEGFP-C1 vector. (**C**) The t-GFP1 DNA was demonstrated. (**D**) The t-GFP2 DNA was demonstrated.

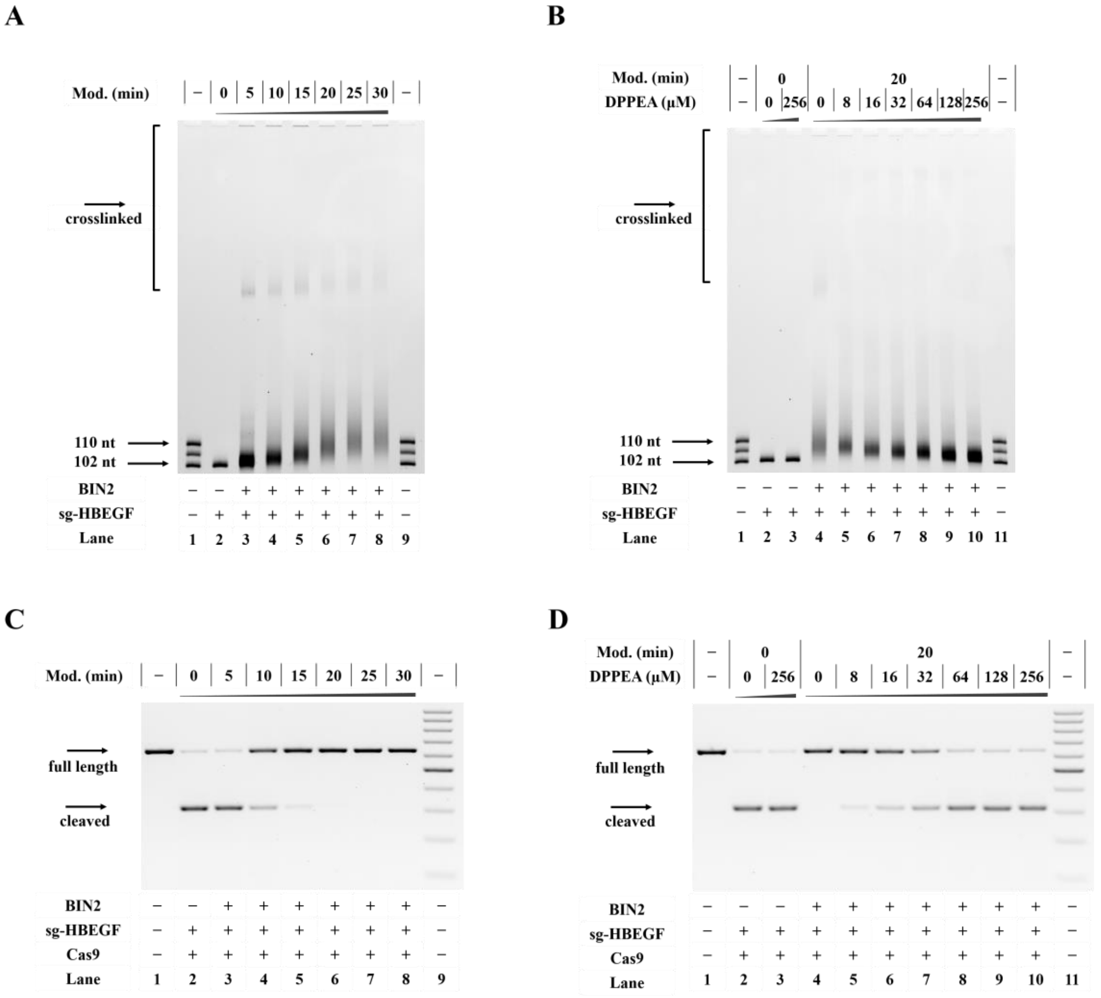

Figure S4. DPPEA activation of crosslinked sg-HBEGF to reversible controlling CRISPR-Cas9. Reactions were conducted as described in the Experimental Section. (A) Gel electrophoretic analysis of crosslinked sg-HBEGF. Lanes 1, 9: RNA marker (sg-HBEGF, R-106 nt, R-110 nt); lane 2: untreated sg-HBEGF; lanes 3–8: crosslinked sg-HBEGF. (B) DPPEA reduction of crosslinked sg-HBEGF. Lanes 1, 11: RNA marker (sg-HBEGF, R-106 nt, R-110 nt); lane 2: untreated sg-HBEGF; lane 3: untreated sg-HBEGF, 256 μM DPPEA; lanes 4–10: crosslinked sg-HBEGF (12.5 mM BIN2, 20 min) treated with DPPEA (0–256 μM). (C) The influence of crosslinked sg-HBEGF on CRISPR-Cas9. Lane 1: intact DNA; lane 2: untreated sg-HBEGF; lanes 3–8: crosslinked sg-HBEGF with different levels; lane 9: DNA marker (100–1000 bp). (D) DPPEA activation of crosslinked sg-HBEGF to recover the cleavage ability of CRISPR-Cas9. Lane 1: intact DNA; lane 2: untreated sg-HBEGF; lane 3: untreated sg-HBEGF, 256 μM DPPEA; lanes 4–10: crosslinked sg-HBEGF (12.5 mM BIN2, 20 min) was treated with DPPEA (0–256 μM); lane 11: DNA marker (100–1000 bp).

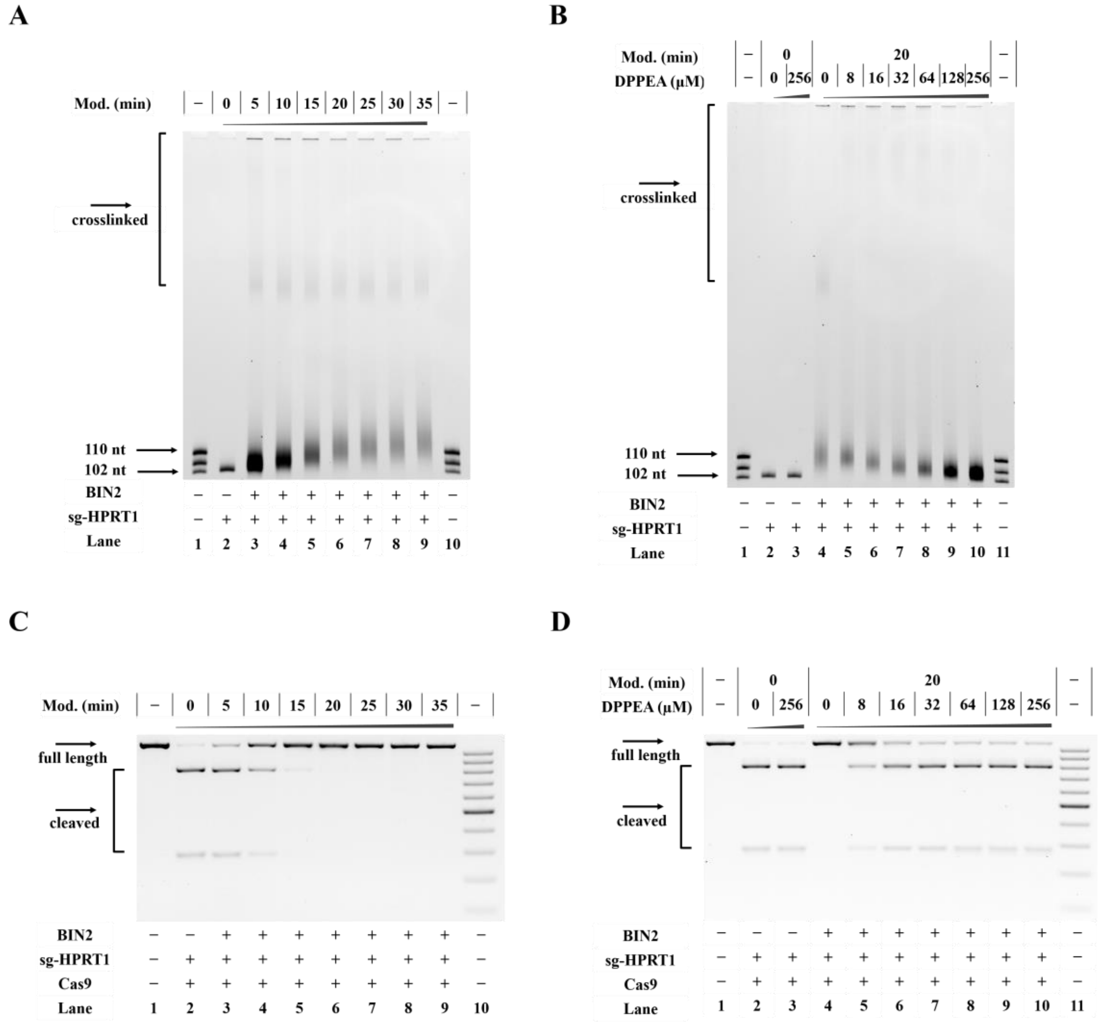

Figure S5. DPPEA activation of crosslinked sg-HPRT1 to reversible controlling CRISPR-Cas9. Reactions were conducted as described in the Experimental Section. (A) Gel electrophoretic analysis of crosslinked sg-HPRT1. Lanes 1, 10: RNA marker (sg-HPRT1, R-106 nt, R-110 nt); lane 2: untreated sg-HPRT1; lanes 3–9: crosslinked sg-HPRT1. (B) DPPEA reduction of crosslinked sg-HPRT1. Lanes 1, 11: RNA marker (sg-HPRT1, R-106 nt, R-110 nt); lane 2: untreated sg-HPRT1; lane 3: untreated sg-HPRT1, 256 μM DPPEA; lanes 4–10: crosslinked sg-HPRT1 (12.5 mM BIN2, 20 min) was treated with DPPEA (0–256 μM). (C) The influence of crosslinked sg-HPRT1 on CRISPR-Cas9. Lane 1: intact DNA; lane 2: untreated sg-HPRT1; lanes 3–9: crosslinked sg-HPRT1 with different levels; lane 10: DNA marker (100–1000 bp). (D) DPPEA activation of crosslinked sg-HPRT1 to recover the cleavage ability of CRISPR-Cas9. Lane 1: intact DNA; lane 2: untreated sg-HPRT1; lane 3: untreated sg-HPRT1, 256 μM DPPEA; lanes 4–10: crosslinked sg-HPRT1 was treated with DPPEA (0–256 μM); lane 11: DNA marker (100–1000 bp).

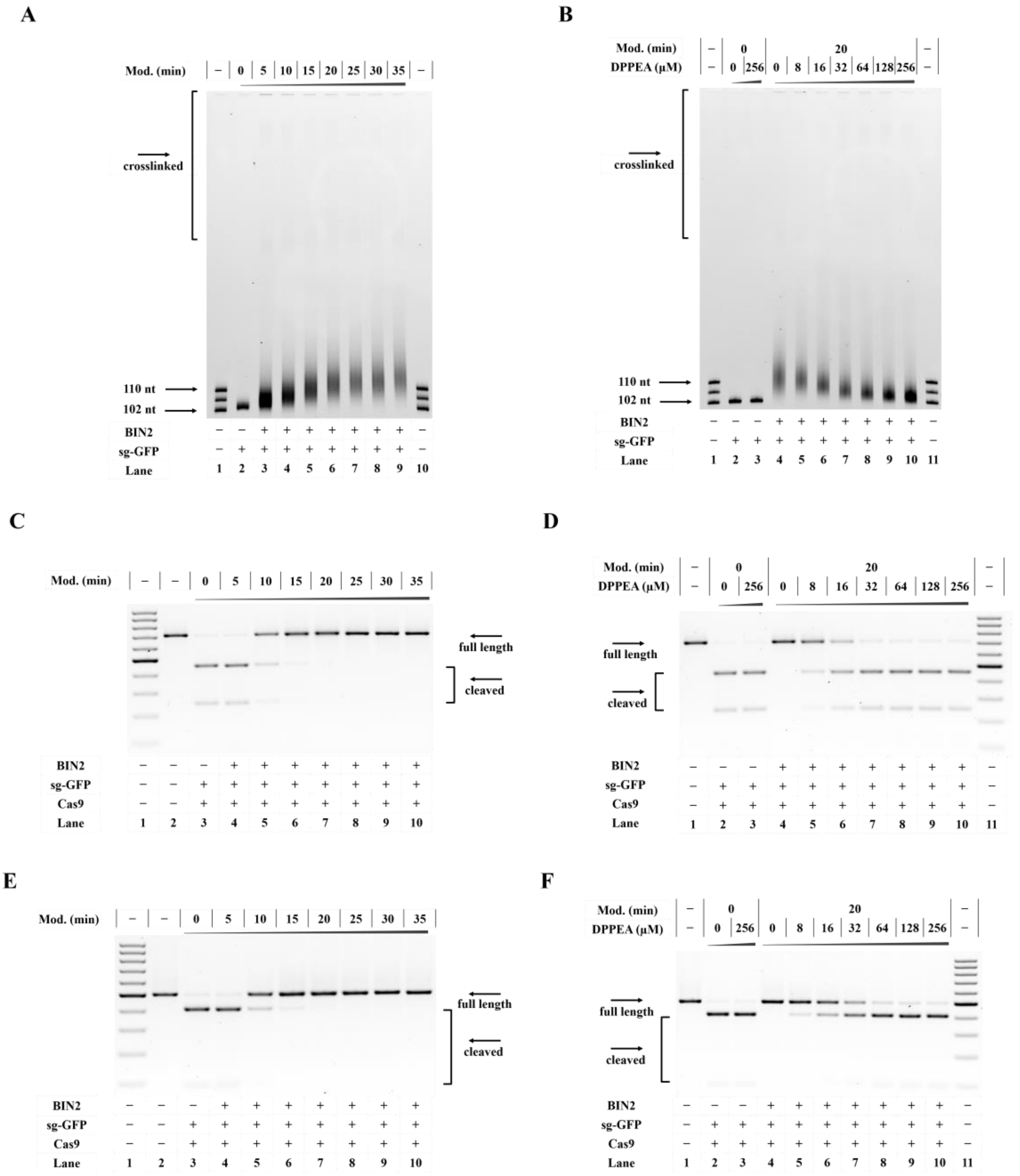

Figure S6. DPPEA activation of crosslinked sg-GFP to reversible controlling CRISPR-Cas9. Reactions were conducted as described in the Experimental Section. (A) Gel electrophoretic analysis of crosslinked sg-GFP. Lanes 1, 10: RNA marker (sg-GFP, R-106 nt, R-110 nt); lane 2: untreated sg-GFP; lanes 3–9: crosslinked sg-GFP. (B) DPPEA reduction of crosslinked sg-GFP. Lanes 1, 11: RNA marker (sg-GFP, R-106 nt, R-110 nt); lane 2: untreated sg-GFP; lane 3: untreated sg-GFP, 256 μM DPPEA; lanes 4–10: crosslinked sg-GFP (12.5 mM BIN2, 20 min) treated with DPPEA (0–256 μM). (C) The influence of crosslinked sg-GFP on CRISPR-Cas9 cleave t-GFP1. Lane 1: DNA marker (100–1000 bp); lane 2: intact t-GFP1; lane 3: untreated sg-GFP; lanes 4–10: crosslinked sg-GFP with different levels. (D) DPPEA activation of crosslinked sg-GFP to restore the cleavage ability of CRISPR-Cas9 to t-GFP1. Lane 1: intact t-GFP1; lane 2: untreated sg-GFP; lane 3: untreated sg-GFP, 256 μM DPPEA; lanes 4–10: crosslinked sg-GFP treated with DPPEA (0–256 μM); lane 11: DNA marker (100–1000 bp). (E) The influence of crosslinked sg-GFP on CRISPR-Cas9 cleave t-GFP2. Lane 1: DNA marker (100–1000 bp); lane 2: intact t-GFP2; lane 3: untreated sg-GFP; lanes 4–10: crosslinked sg-GFP with different levels. (F) DPPEA activation of crosslinked sg-GFP to restore the cleavage ability of CRISPR-Cas9 to t-GFP2. Lane 1: intact t-GFP2; lane 2: untreated sg-GFP;

lane 3: untreated sg-GFP, 256 μM DPPEA; lanes 4–10: crosslinked sg-GFP was treated with DPPEA (0–256 μM); lane 11: DNA marker (100–1000 bp).

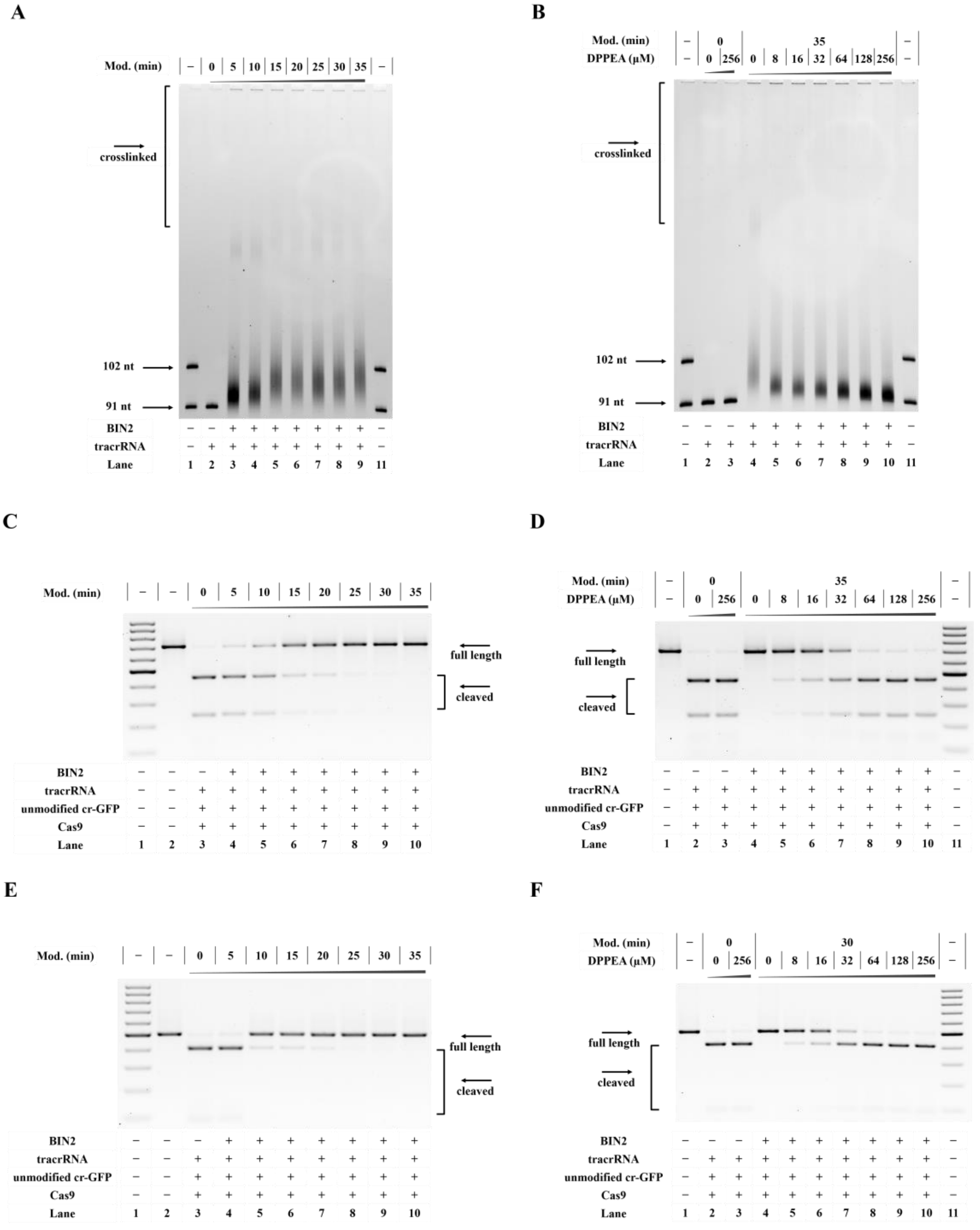

Figure S7. DPPEA activation of crosslinked tracrRNA and unmodified cr-GFP to reversible controlling CRISPR-Cas9. Reactions were conducted as described in the Experimental Section. (A) Gel electrophoretic analysis of crosslinked tracrRNA. Lanes 1, 11: RNA marker (tracrRNA, R-102 nt); lane 2: untreated tracrRNA; lanes 3–9: crosslinked tracrRNA (12.5 mM BIN2, various times). (B) DPPEA reduction of crosslinked tracrRNA. Lanes 1, 11: RNA marker (tracrRNA, R-102 nt); lane 2: untreated tracrRNA; lane 3: untreated tracrRNA, 256 μM DPPEA; lanes 4–10: crosslinked tracrRNA (12.5 mM BIN2, 35 min) treated with DPPEA (0–256 μM). (C) The influence of crosslinked tracrRNA paired with cr-GFP on CRISPR-Cas9 cleave t-GFP1. Lane 1: DNA marker (100–1000 bp); lane 2: intact t-GFP1; lane 3: untreated tracrRNA; lanes 4–10: crosslinked tracrRNA with different levels. (D)

DPPEA activation of crosslinked tracrRNA paired with cr-GFP to restore the cleavage ability of CRISPR-Cas9 to t-GFP1. Lane 1: intact t-GFP1; lane 2: untreated tracrRNA; lane 3: untreated tracrRNA, 256 μM DPPEA; lanes 4–10: crosslinked tracrRNA (12.5 mM BIN2, 35 min) treated with DPPEA (0–256 μM); lane 11: DNA marker (100–1000 bp). (E) The influence of crosslinked tracrRNA paired with cr-GFP on CRISPR-Cas9 cleave t-GFP2. Lane 1: DNA marker (100–1000 bp); lane 2: intact t-GFP2; lane 3: untreated tracrRNA; lanes 4–10: crosslinked tracrRNA with different levels. (F) DPPEA activation of crosslinked tracrRNA paired with cr-GFP to restore the cleavage ability of CRISPR-Cas9 to t-GFP2. Lane 1: intact t-GFP2; lane 2: untreated tracrRNA; lane 3: untreated tracrRNA, 256 μM DPPEA; lanes 4–10: crosslinked tracrRNA (12.5 mM BIN2, 35 min) paired with cr-GFP were treated with DPPEA (0–256 μM); lane 11: DNA marker (100–1000 bp).

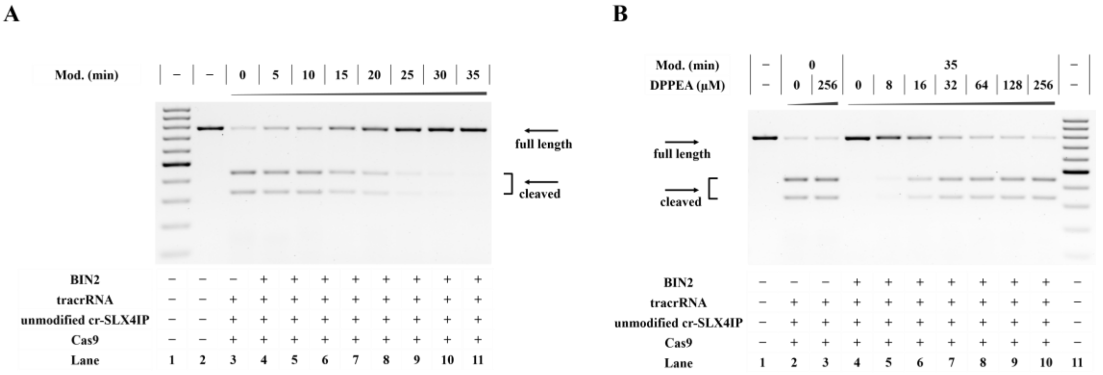

Figure S8. DPPEA activation of crosslinked tracrRNA and unmodified cr-SLX4IP to reversible regulating CRISPR-Cas9. Reactions were conducted as described in the Experimental Section. (A) The influence of crosslinked tracrRNA paired with cr-SLX4IP on CRISPR-Cas9 cleave target DNA. Lane 1: DNA marker (100–1000 bp); lane 2: intact DNA; lane 3: untreated tracrRNA paired with cr-SLX4IP; lanes 4–10: crosslinked tracrRNA (12.5 mM, 5–35 min) paired with cr-SLX4IP. (B) DPPEA activation of crosslinked tracrRNA paired with cr-SLX4IP to restore the cleavage ability of CRISPR-Cas9 to target DNA. Lane 1: intact DNA; lane 2: untreated tracrRNA; lane 3: untreated tracrRNA, 256 μM DPPEA; lanes 4–10: crosslinked tracrRNA (12.5 mM BIN2, 35 min) paired with cr-SLX4IP were treated with DPPEA (0–256 μM); lane 11: DNA marker (100–1000 bp).

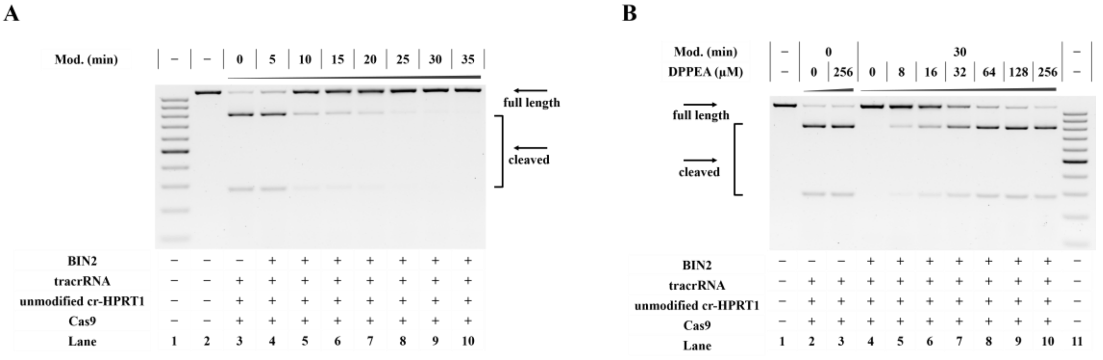

Figure S9. DPPEA activation of crosslinked tracrRNA and unmodified cr-HPRT1 to reversible regulating CRISPR-Cas9. Reactions were conducted as described in the Experimental Section. (A) The influence of crosslinked tracrRNA paired with cr-HPRT1 on CRISPR-Cas9 cleave target DNA. Lane 1: DNA marker (100–1000 bp); lane 2: intact DNA; lane 3: untreated tracrRNA paired with cr-HPRT1; lanes 4–10: crosslinked tracrRNA (12.5 mM, 5–35 min) paired with cr-HPRT1. (B) DPPEA activation of crosslinked tracrRNA paired with cr-HPRT1 to restore the cleavage ability of CRISPR-Cas9 to target DNA. Lane 1: intact DNA; lane 2: untreated tracrRNA; lane 3: untreated tracrRNA, 256 μM DPPEA; lanes 4–10: crosslinked tracrRNA (12.5 mM BIN2, 35 min) paired with cr-

HPRT1 were treated with DPPEA (0–256 μM); lane 11: DNA marker (100–1000 bp).

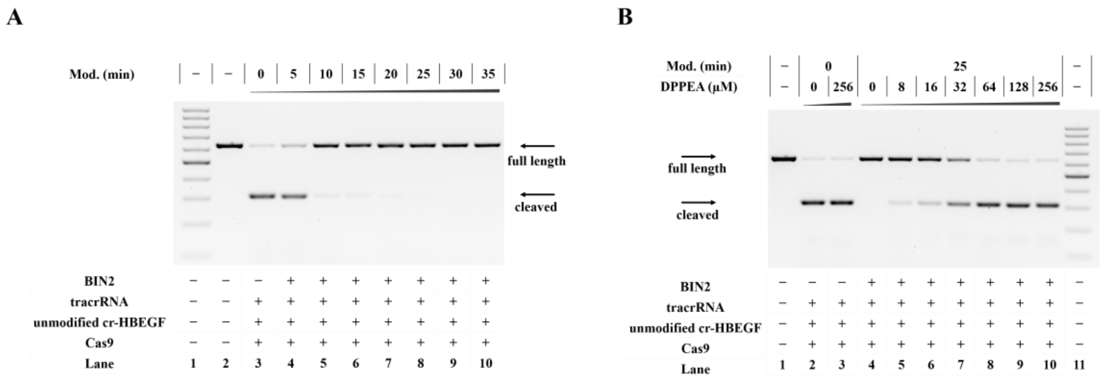

Figure S10. DPPEA activation of crosslinked tracrRNA and unmodified cr-HBEGF to reversible controlling CRISPR-Cas9. Reactions were conducted as described in the Experimental Section. (A) The influence of crosslinked tracrRNA paired with cr-HBEGF on CRISPR-Cas9 cleave target DNA. Lane 1: DNA marker (100–1000 bp); lane 2: intact DNA; lane 3: untreated tracrRNA paired with cr-HBEGF; lanes 4–10: crosslinked tracrRNA (12.5 mM BIN2, 5–35 min) paired with cr-HBEGF. (B) DPPEA activation of crosslinked tracrRNA paired with cr-HBEGF to restore the cleavage ability of CRISPR-Cas9 to target DNA. Lane 1: intact DNA; lane 2: untreated tracrRNA; lane 3: untreated tracrRNA, 256 μM DPPEA; lanes 4–10: crosslinked tracrRNA (12.5 mM BIN2, 25 min) paired with cr-HPRT1 were treated with DPPEA (0–256 μM); lane 11: DNA marker (100–1000 bp).

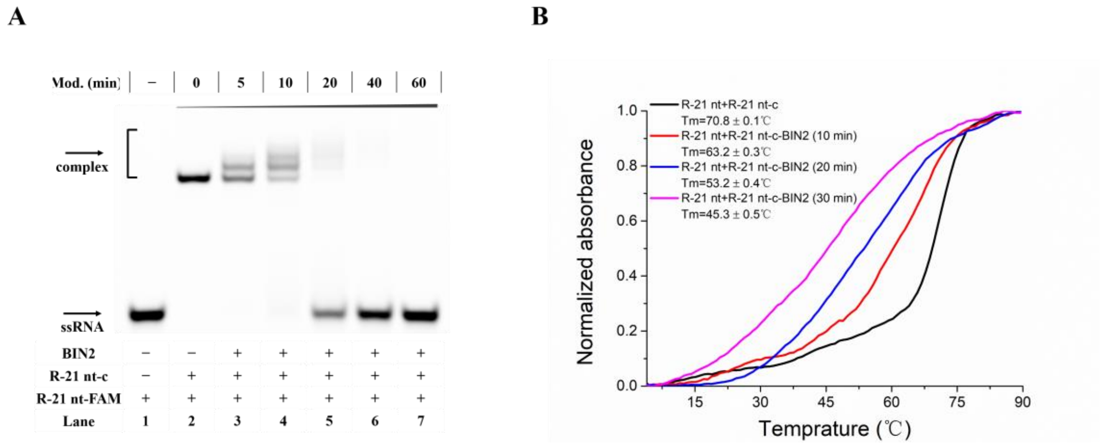

Figure S11. Crosslinking strategy for regulating RNA-RNA interaction. Reactions were performed as described in the Experimental Section. Time-dependent inhibition of RNA-RNA hybridization. (A) Lane 1: Untreated R-21nt-c-FAM; lanes 2–7: the time course of inhibition of crosslinked RNA (12.5 mM BIN2, 0–60 min) and complementary RNA. (B) Crosslinked RNA (12.5 mM BIN2, 0–30 min) inhibition of the binding to complementary RNA.

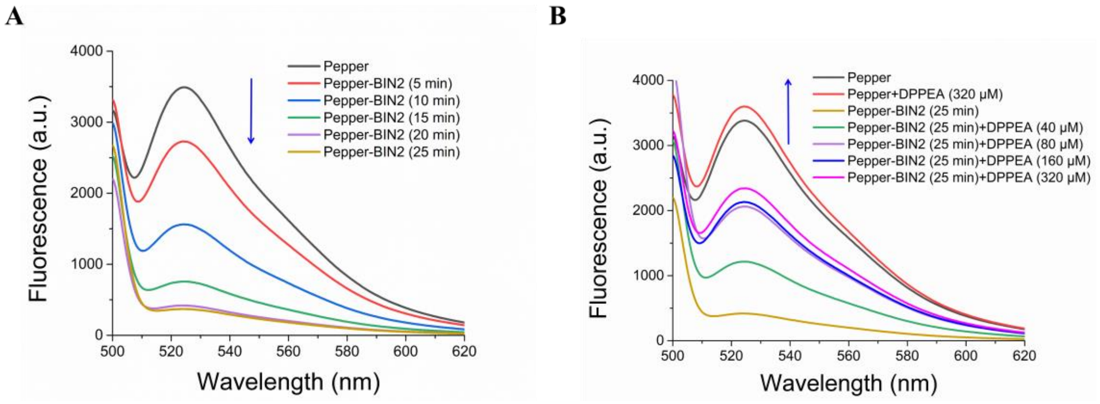

Figure S12. Crosslinking strategy for controlling RNA folding. Reactions were performed as described in the Experimental Section. Time-dependent inhibition of crosslinked Peppers binding to HBC525 fluorophores. (A) Crosslinked Peppers (12.5 mM BIN2, 0–25 min) lower the binding ability to HBC525. (B) Treatment with DPPEA (0–320 μM) to recover Fluorescence signal.

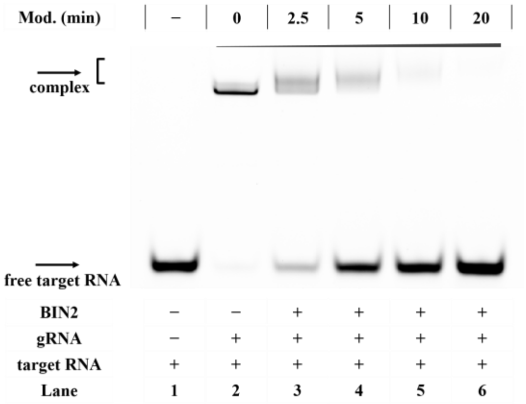

Figure S13. Crosslinking strategy for regulating RNA hybridization. Reactions were performed as described in the Experimental Section. Time-dependent inhibition of RNA-RNA hybridization. Lane 1: target RNA (5′ ends with FAM labeling); lane 2: untreated gRNA with target RNA; lanes 3–6: time-dependent inhibition of crosslinked gRNA (12.5 mM BIN2, 0–20 min) to target RNA hybridization.

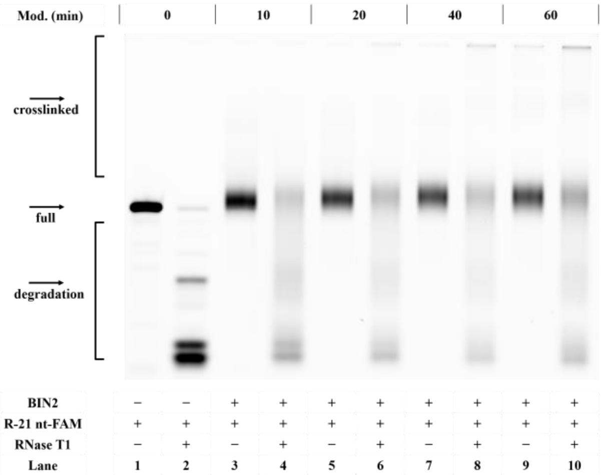

Figure S14. Crosslinking strategy protects RNA from RNase T1 degradation. Reactions were performed as described in the Experimental Section. Time-dependent inhibition of RNA degradation by RNase T1. Lane 1: Untreated R-21nt-c-FAM; lane 2: R-21nt-c-FAM with RNase T1 on ice for 10 min; lanes 3, 5, 7, 9: crosslinked R-

21nt-c-FAM (12.5 mM BIN2; 10, 20, 40, 60 min); lanes 4, 6, 8, 10: crosslinked R-21nt-c-FAM (12.5 mM BIN2; 10, 20, 40, 60 min) incubation with RNase T1 on ice for 10 min.

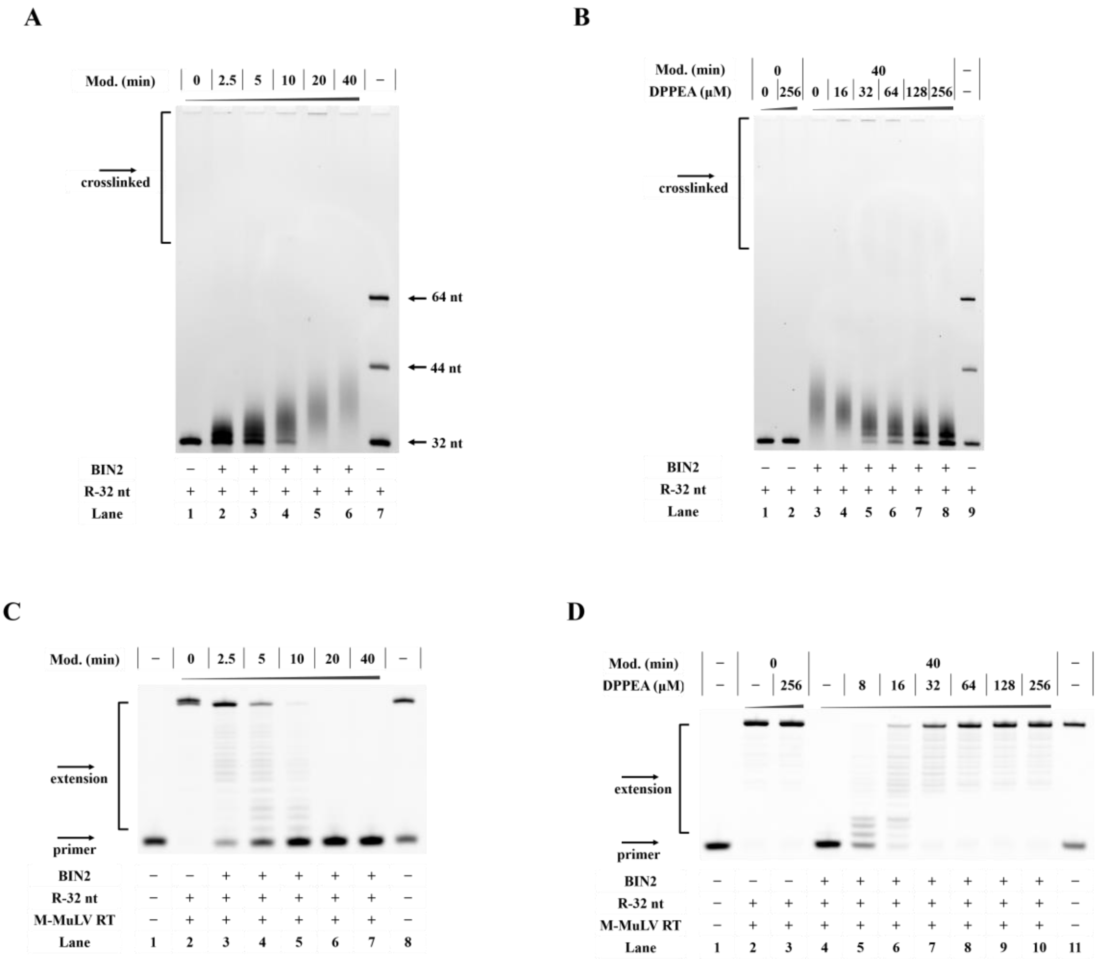

Figure S15. DPPEA activation of crosslinked RNA template to reversible regulating primer elongation. Reactions were conducted as described in the Experimental Section. (A) Gel electrophoretic analysis of crosslinked RNA template (R-32 nt). Lane 1: Untreated RNA template (R-32 nt); lanes 2–6: crosslinked R-32 nt (12.5 mM BIN2, 2.5–40 min); lane 7: RNA marker (R-32nt, R-44 nt, R-64 nt). (B) DPPEA reduction of crosslinked RNA to activation of primer elongation. Lane 1: Untreated R-32 nt; lane 2: untreated R-32 nt, 256 μM DPPEA; lanes 3–8: crosslinked R-32 nt (12.5 mM BIN2, 40 min) was treated with DPPEA (0–256 μM); lane 9: RNA marker (R-32 nt, R-44 nt, R-64 nt). (C) The influence of crosslinked R-32 nt on primer elongation. Lane 1: DNA primer; lane 2: untreated R-32 nt; lanes 3–7: crosslinked R-32 nt with different levels; lane 8: DNA marker (D-11 nt, D-32 nt). (D) DPPEA reduction of crosslinked RNA-32 nt to activation of primer elongation. Lane 1: DNA primer; lane 2: untreated R-32 nt; lane 3: untreated R-32 nt, 256 μM DPPEA; lanes 4–10: crosslinked R-32 nt was treated with DPPEA (0–256 μM); lane 11: DNA marker (D-11 nt, D-32 nt).

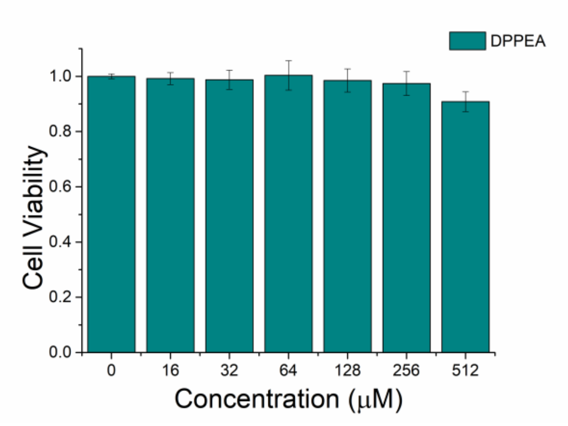

Figure S16. The influence of DPPEA to Hela-OC viability. The toxicity of DPPEA was measured by MTT assay on Hela-OC cells. The value of DMEM control was set to 100% (seem as relative growth). All values were calculated as the means ± SEM from three independent experiments. Error bars: standard deviation of the means of triplicate samples, and the result showed that the cytotoxicity of DPPEA was over 512 μM.

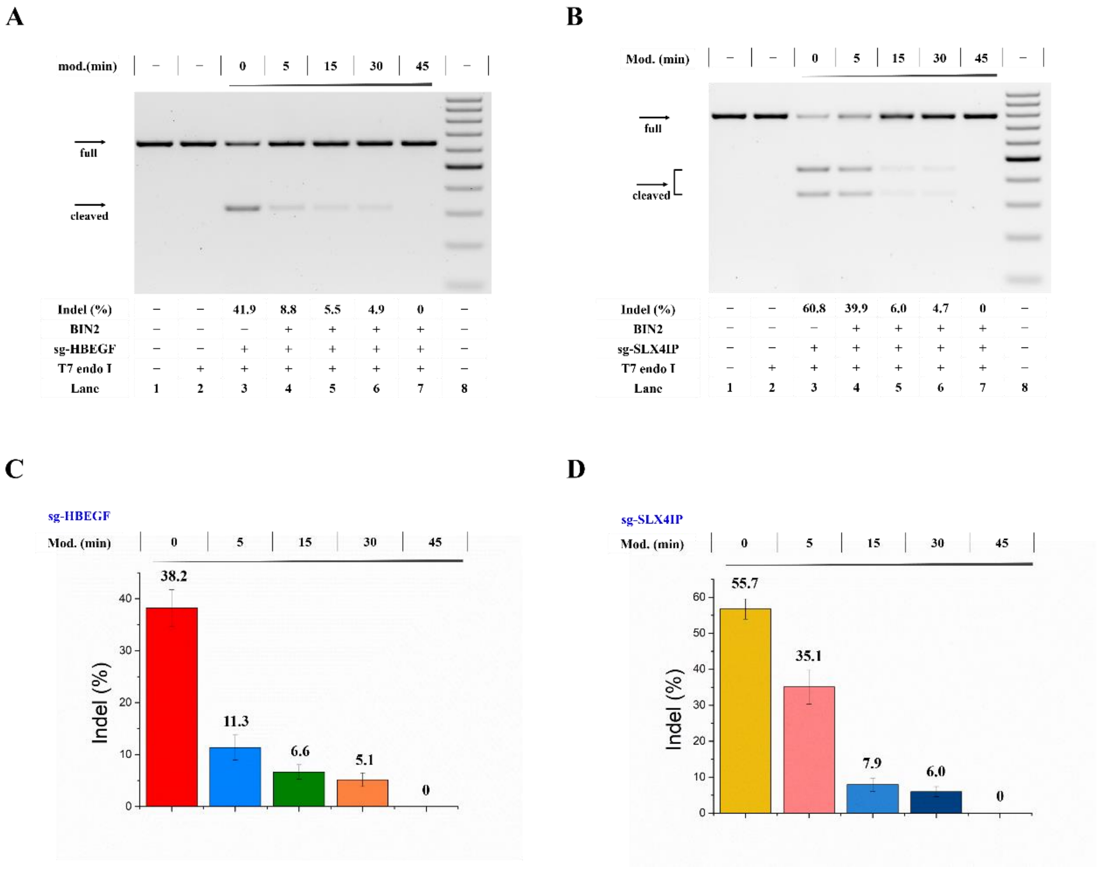

Figure S17. Crosslinking strategy controlling gene editing in Hela-OC cells. Cellular studies were conducted as described in the Experimental Section. Two types of sgRNAs were transfected into Hela-OC cells. After incubation of 24 h, the efficiency of gene editing rates (insertion/deletion mutation) was assessed by T7EI nuclease assay. All samples were tested in three independent experiments. (A) Time-dependent inhibition of gene editing by crosslinked sg-HBEGF. Lane 1: target control; lane 2: no sg-HBEGF control; lane 3: untreated sg-HBEGF; lanes 4–7: crosslinked sg-HBEGF (5–45 min); lane 8: DNA marker (100–1000 bp). (B) Time-dependent inhibition of gene editing by crosslinked sg-SLX4IP. Lane 1: target control; lane 2: no sg-SLX4IP control; lane 3: untreated sg-SLX4IP;

lanes 4–7: crosslinked sg-SLX4IP (5–45 min); lane 8: DNA marker (100–1000 bp). (C) Crosslinked sg-HBEGF inhibition of gene editing was depicted in a bar graph with error lines plotted. (D) Crosslinked sg-SLX4IP inhibition of gene editing was depicted in a bar graph with error lines plotted.

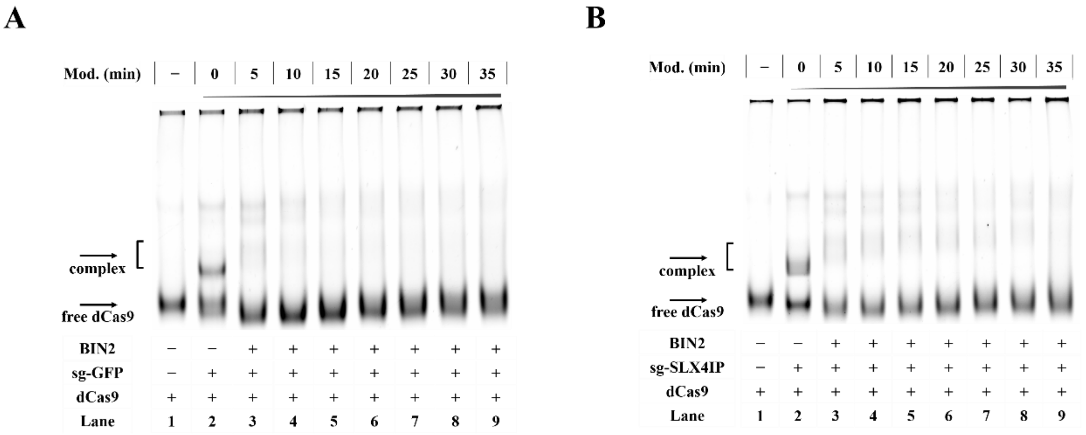

Figure S18. Crosslinking strategy for controlling the interaction between RNA and protein. Reactions were performed as described in the Experimental Section. Time-dependent inhibited the formation of RNA-protein complex. (A) Lane 1: dCas9 (with Halo ligand labeling); lane 2: untreated sg-GFP; lanes 3–9: time-dependent inhibition of crosslinked sg-GFP (12.5 mM BIN2, 5–35 min) to formation duplex. (B) Lane 1: dCas9 (with Halo ligand labeling); lane 2: untreated sg-SLX4IP; lanes 3–9: time-dependent inhibition of crosslinked sg-SLX4IP (12.5 mM BIN2, 5–35 min) to formation duplex.

Appendix A: NMR spectra

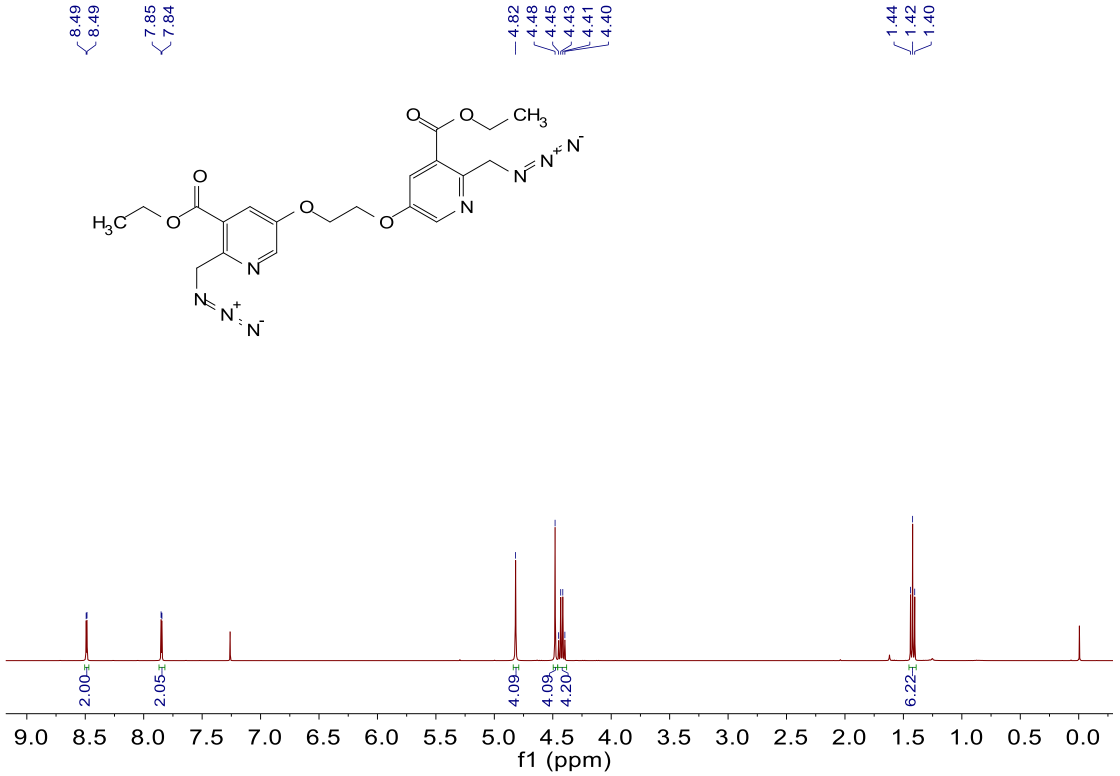

^{1}H NMR (400 MHz, $CDCl_3$) spectrum of compound **2**.

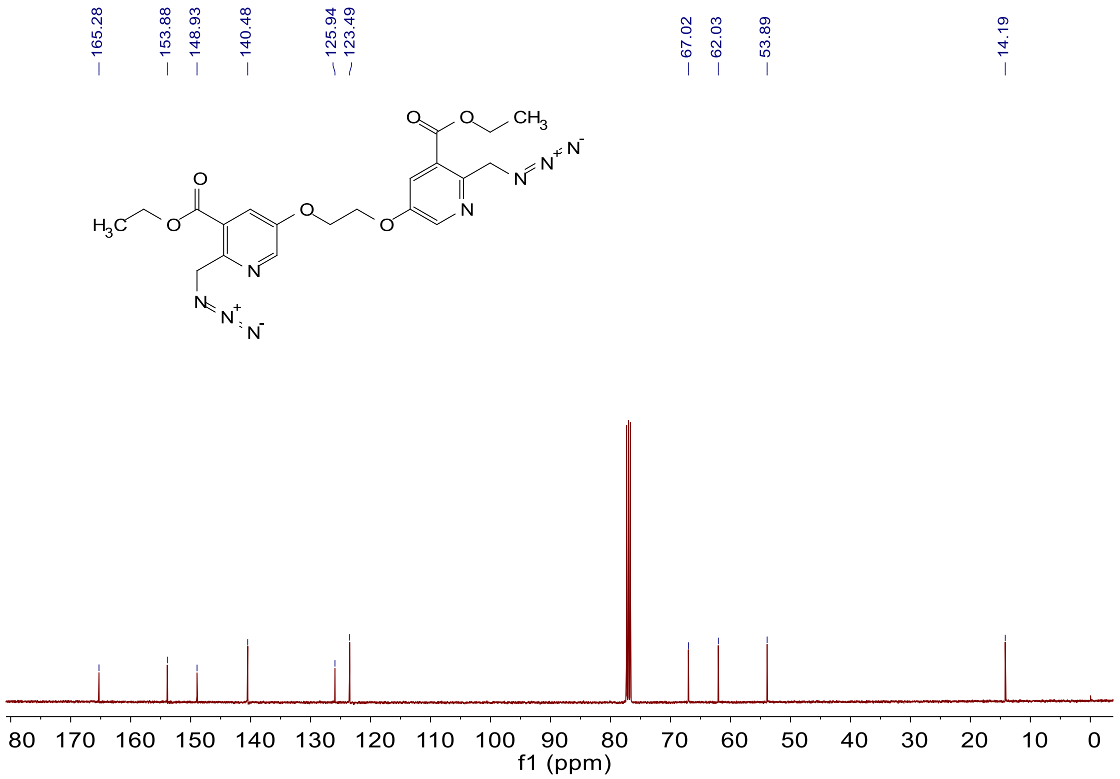

^{13}C NMR (101 MHz, $CDCl_3$) spectrum of compound **2**.

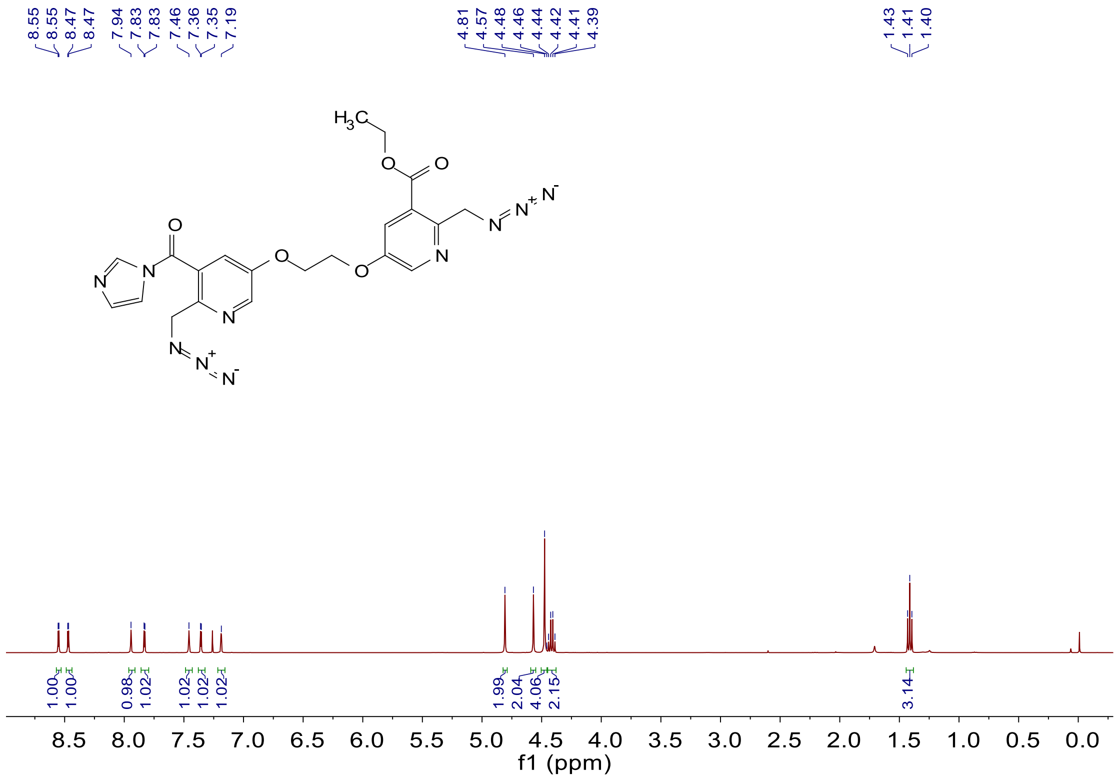

^{1}H NMR (400 MHz, $CDCl_3$) spectrum of compound **3 (BIN-C)**.

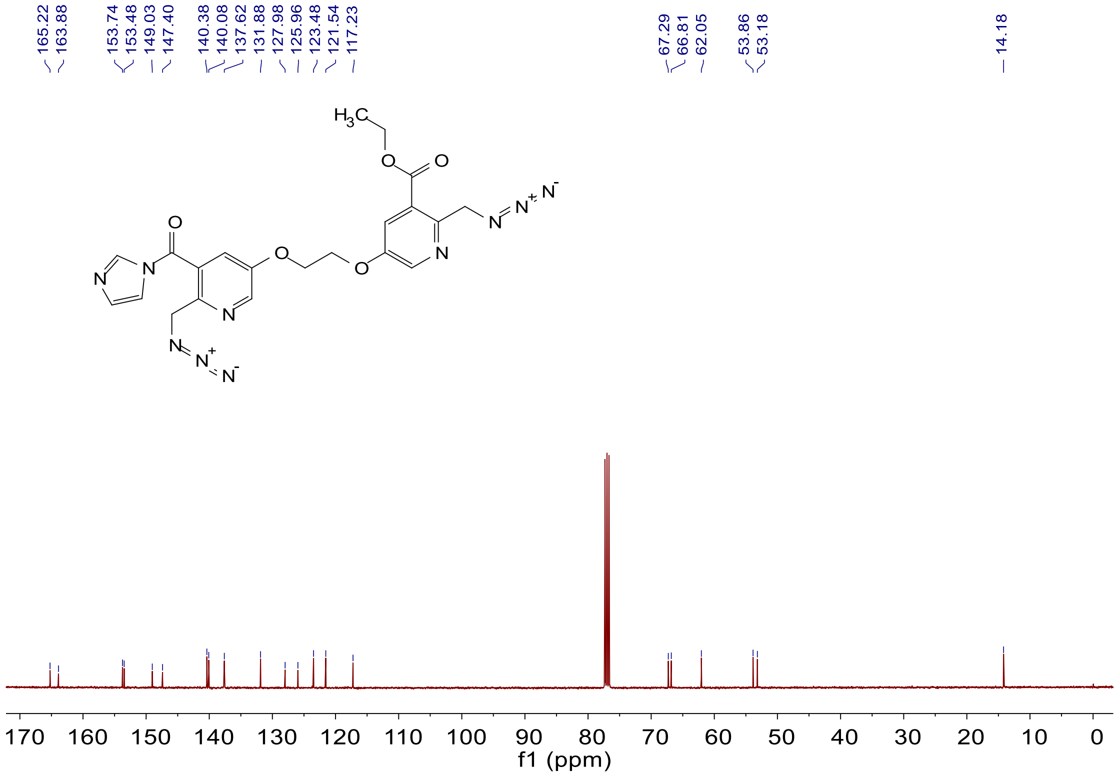

^{13}C NMR (101 MHz, $CDCl_3$) spectrum of compound **3 (BIN-C)**.

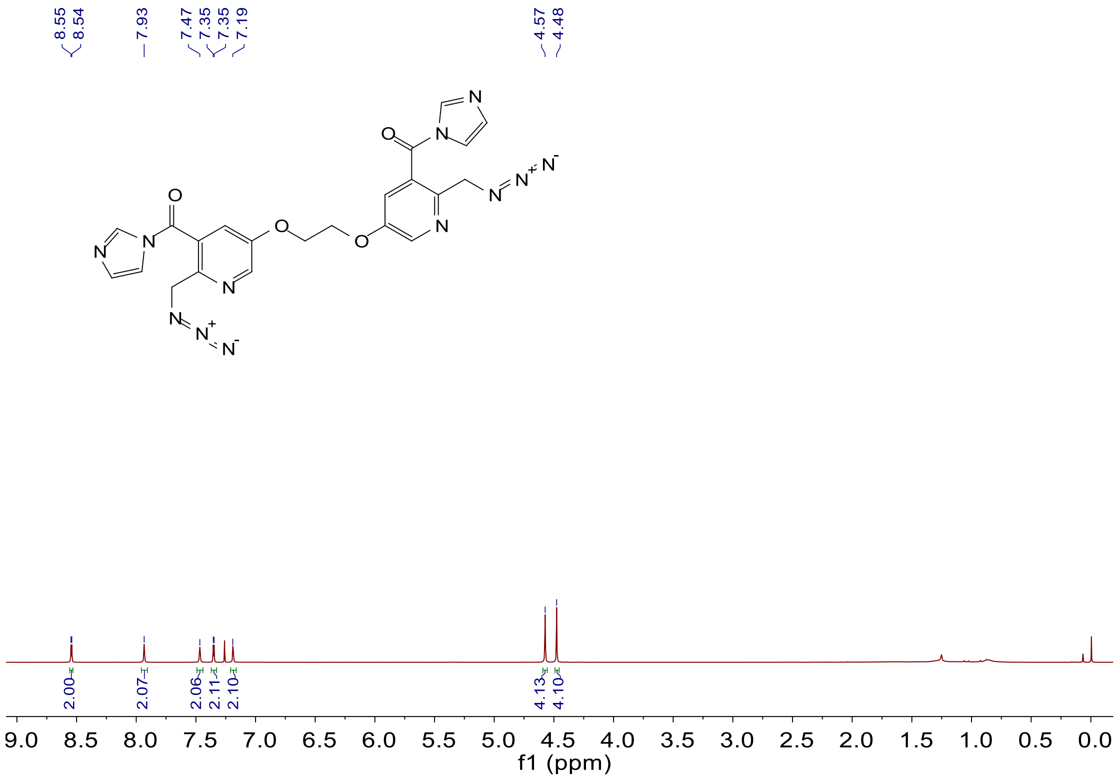

^{1}H NMR (400 MHz, $CDCl_3$) spectrum of compound **4 (BIN2)**.

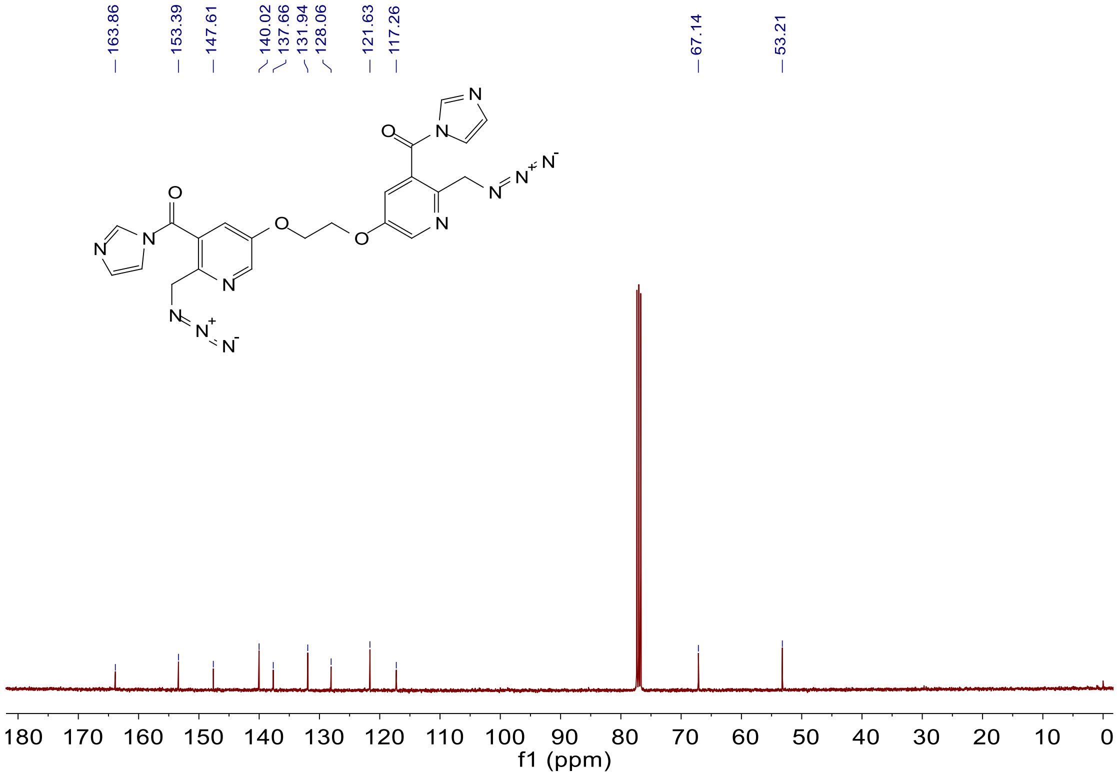

^{13}C NMR (101 MHz, $CDCl_3$) spectrum of compound **4 (BIN2)**.

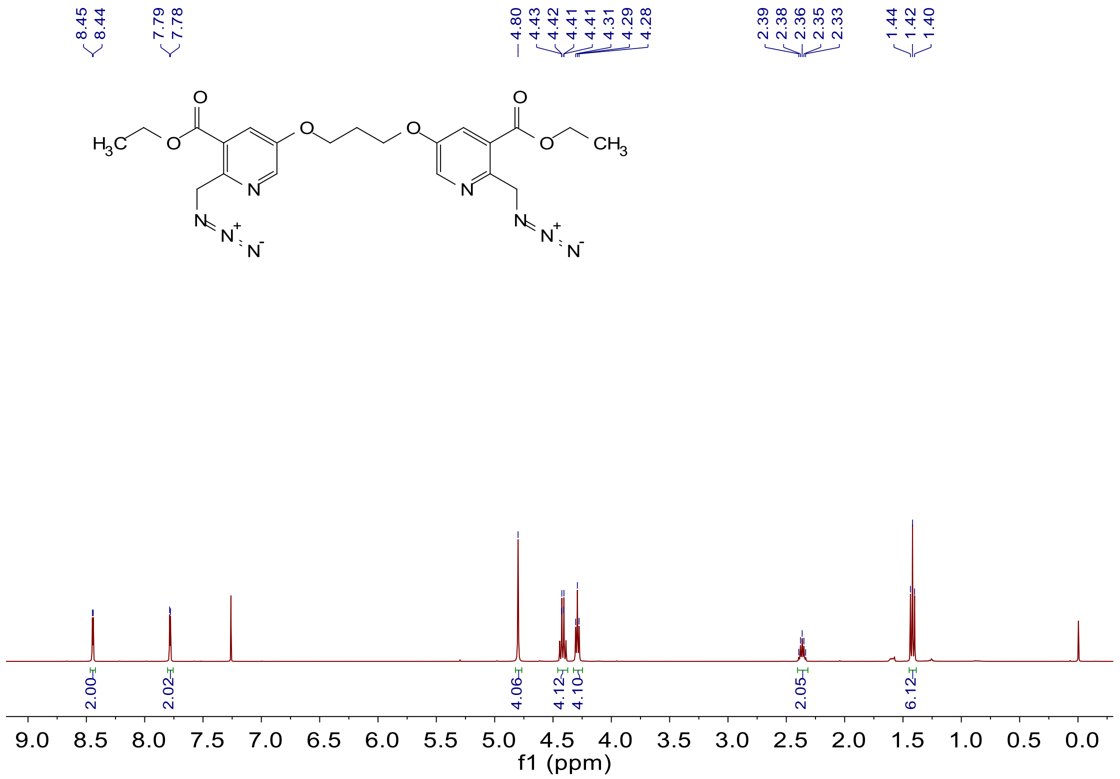

^{1}H NMR (400 MHz, $CDCl_3$) spectrum of compound **5**.

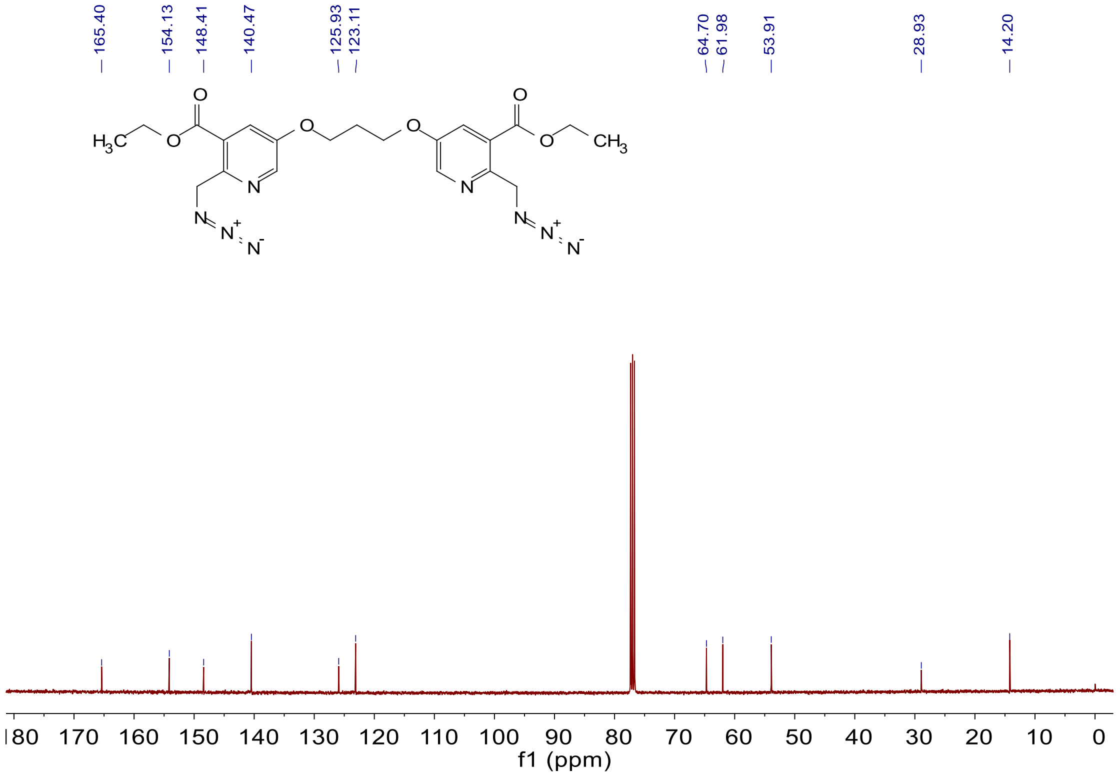

^{13}C NMR (101 MHz, $CDCl_3$) spectrum of compound **5**.

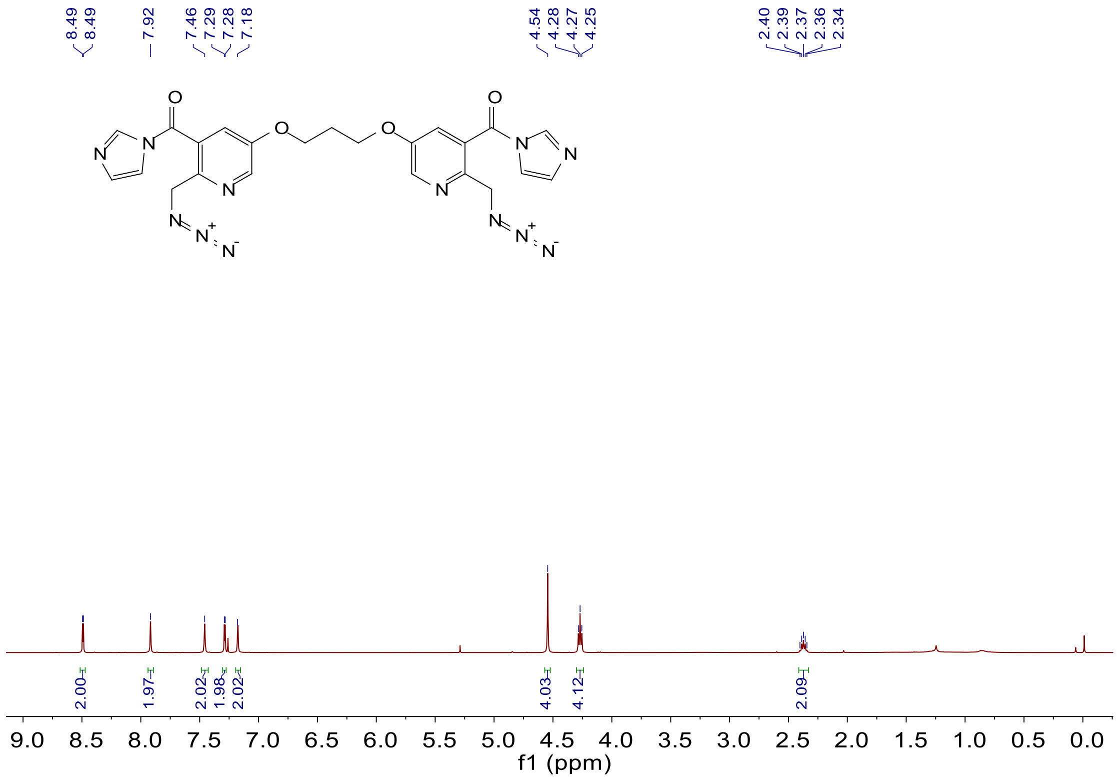

^{1}H NMR (400 MHz, $CDCl_3$) spectrum of compound **6 (BIN-3)**.

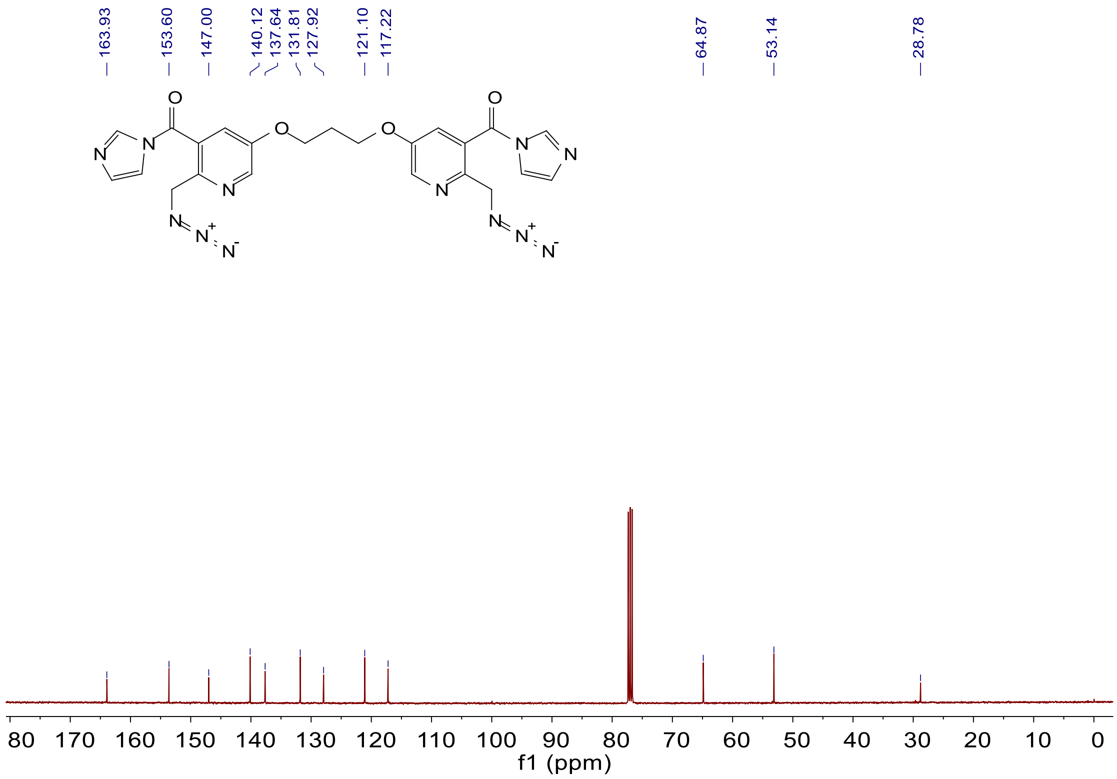

^{13}C NMR (101 MHz, $CDCl_3$) spectrum of compound **6 (BIN-3)**.

Appendix B: HRMS spectra of the key compounds in the current study

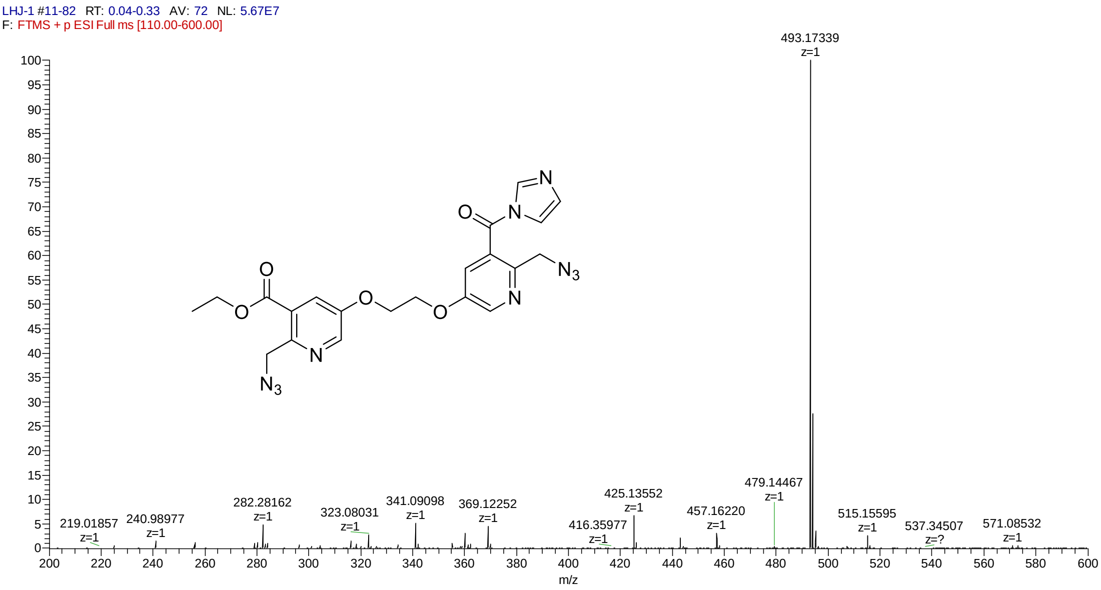

HRMS spectrum of compound 3 (BIN-C)

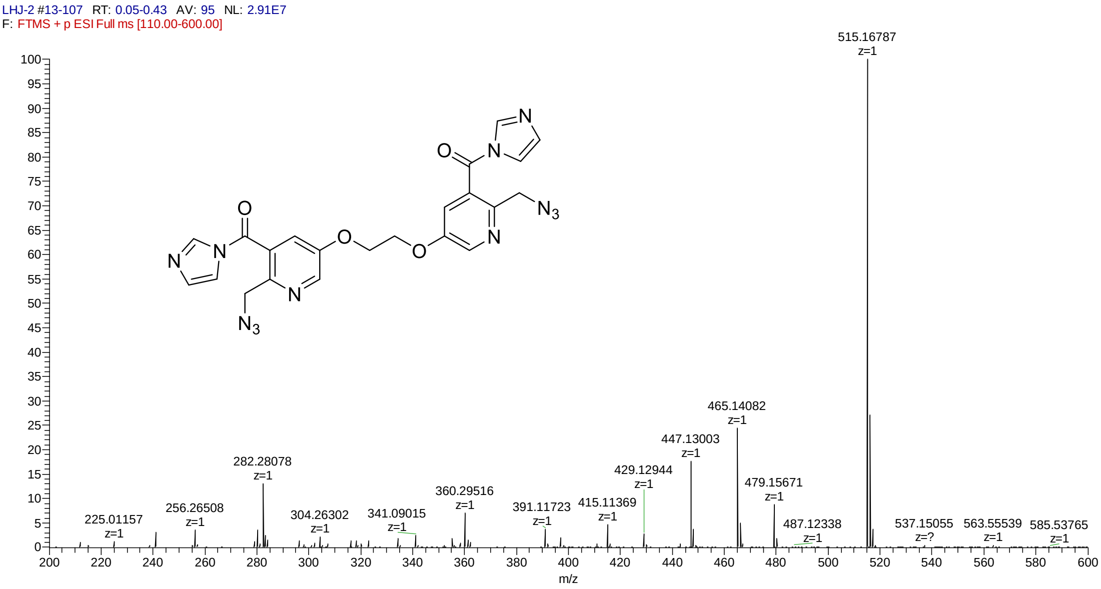

HRMS spectrum of compound 4 (BIN2)

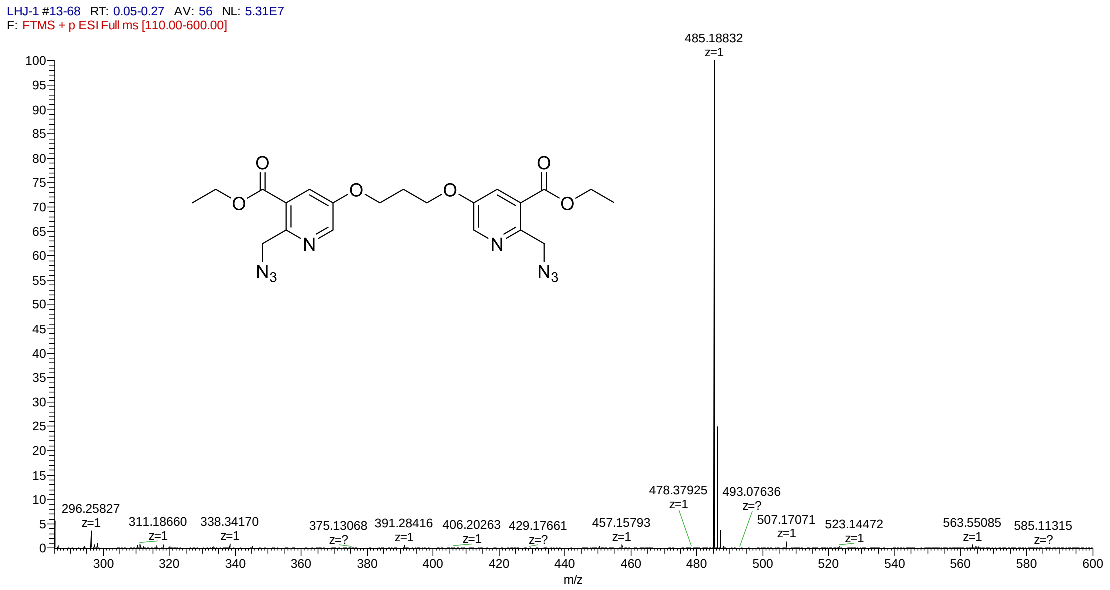

HRMS spectrum of compound 5

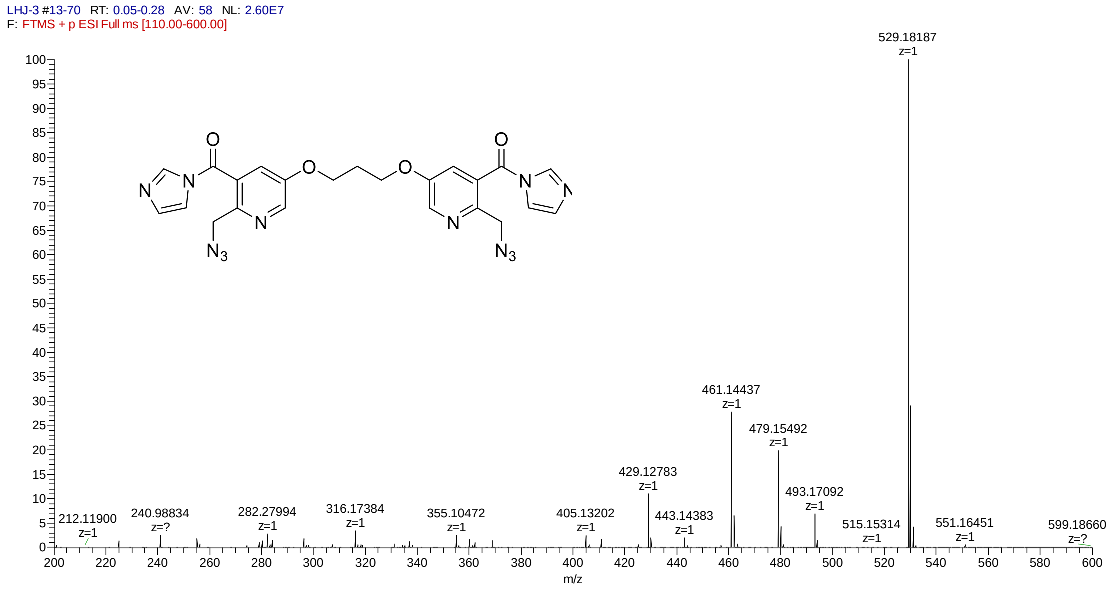

HRMS spectrum of compound 6 (BIN-3)

References

[1] W. A. Velema, H. S. Park, A. Kadina, L. Orbai, E. T. Kool, *Angew. Chem. Int. Ed.* **2020**, *59*, 22017-22022.

[2] X. Chen, D. Zhang, N. Su, B. Bao, X. Xie, F. Zuo, L. Yang, H. Wang, L. Jiang, Q. Lin, M. Fang, N. Li, X. Hua, Z. Chen, C. Bao, J. Xu, W. Du, L. Zhang, Y. Zhao, L. Zhu, J. Loscalzo, Y. Yang, *Nat. Biotechnol.* **2019**, *37*, 1287-1293.

[3] a) S. R. Wang, L. Y. Wu, H. Y. Huang, W. Xiong, J. Liu, L. Wei, P. Yin, T. Tian, X. Zhou, *Nat. Commun.* **2020**, *11*, 91; b) W. Xiong, X. Y. Liu, Q. Q. Qi, H. M. Ji, F. B. Liu, C. Zhong, S. M. Liu, T. Tian, X. Zhou, *Nucleic Acids Res.* **2022**, *50*, 1241-1255.
